# Targeting LINC01711 in FAP^+^ cancer-associated fibroblasts overcomes lactate-mediated immunosuppression and enhances anti-PD-1 efficacy in lung adenocarcinoma

**DOI:** 10.1038/s41419-025-07974-6

**Published:** 2025-08-25

**Authors:** Qinglin Wang, Yuxiang Sun, Jianyu Li, Zhizong Li, Fangwei Yuan, Zhijun Xia, Fanchen Meng, Ziyang Shen, Yiyang Shen, Lin Xu, Jie Wang, Xi Chen, Tongyan Liu, Rong Yin

**Affiliations:** 1 Department of Thoracic Surgery, Jiangsu Cancer Hospital, Jiangsu Institute of Cancer Research, Nanjing Medical University Affiliated Cancer Hospital, Nanjing, 210009 China; 2 Jiangsu Key Laboratory of Innovative Cancer Diagnosis & Therapeutics, Nanjing Medical University Affiliated Cancer Hospital, Cancer Institute of Jiangsu Province, Nanjing, 210009 China; 3 The Second People’s Hospital of Changzhou, The Third Affiliated Hospital of Nanjing Medical University, Changzhou Medical Center, Nanjing Medical University, Changzhou, 213003 China; 4 Jiangsu Biobank of Clinical Resources, Nanjing, 210009 China; 5 Nanjing Drum Tower Hospital Center of Molecular Diagnostic and Therapy, State Key Laboratory of Pharmaceutical Biotechnology, Jiangsu Engineering Research Center for MicroRNA Biology and Biotechnology, NJU Advanced Institute of Life Sciences (NAILS), School of Life Sciences, Nanjing University, Nanjing, 210023 China; 6 Collaborative Innovation Center for Cancer Personalized Medicine, Jiangsu Key Lab of Cancer Biomarkers, Prevention and Treatment, Nanjing Medical University, 211116 Nanjing, PR China

**Keywords:** Non-small-cell lung cancer, Cancer microenvironment, Cancer immunotherapy

## Abstract

The limited response rate to immune checkpoint inhibitors (ICIs) remains a significant challenge in the treatment of lung adenocarcinoma (LUAD). In our study, we identified a lactate-based chemical barrier surrounding FAP^+^ cancer-associated fibroblasts (CAFs) within the LUAD microenvironment (TME), which may hinder the infiltration and function of CD8^+^ T cells. Further investigation revealed that FAP^+^ CAFs specifically overexpress LINC01711, which drives lactate production by promoting FGFR1-mediated phosphorylation of lactic dehydrogenase A (LDHA) at the Y10 site and facilitating the formation of active LDHA tetramers. These FAP^+^ CAFs then export lactate into TME via the MCT4 transporter, thereby establishing a chemical barrier and fostering an immunosuppressive TME. Notably, we developed a small extracellular vesicle (sEV)-based in vivo self-assembled siRNA system for in vivo knockdown of LINC01711 and demonstrated its potential to enhance the response rate to ICIs in LUAD. Our findings underscore the pivotal role of FAP^+^ CAFs in driving resistance to ICIs and propose novel therapeutic strategies to overcome this obstacle.

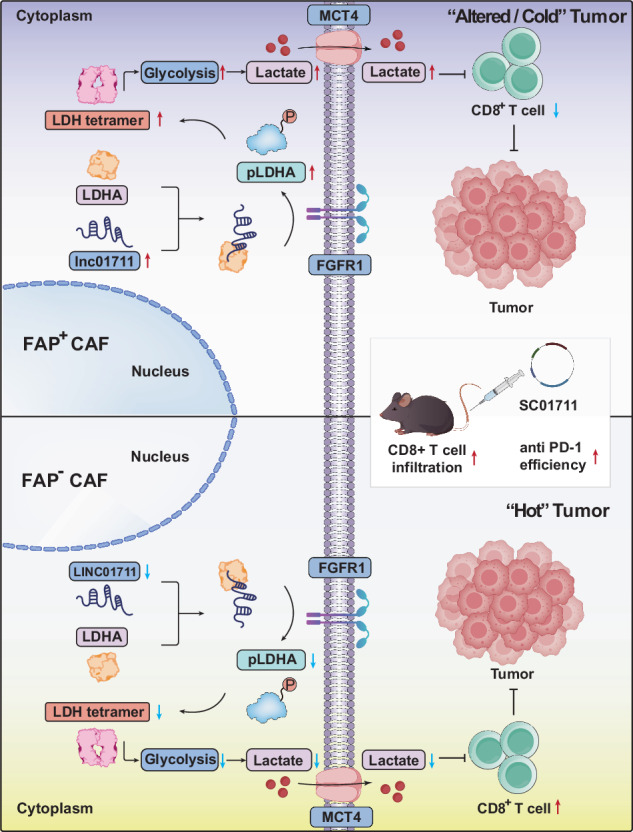

## Introduction

Following the reporting of the KEYNOTE-189 and -407 trials, combining pembrolizumab with chemotherapy were demonstrated improved overall survival (OS) and progression-free survival (PFS) in the first-line treatment of advanced non-small cell lung cancer (NSCLC) [1, 2]. While immune checkpoint inhibitors (ICIs) targeting the PD-1 or CTLA-4 have achieved great success in the field of lung cancer in recent years, the efficacy of ICIs remains suboptimal, with only a fraction of patients responding positively and benefit from ICIs [3–5]. Meanwhile, most responding patients developed acquire resistance to ICIs eventually [6]. The low response rate to ICIs may be associated with “cold” tumor subtype in the majority of patients. In contrast, “hot” tumors, which are highly immunogenic, demonstrate a signature of antitumor CD8^+^ T cell responses, correlating with better clinical responses to ICIs [7]. However, in immunosuppressive “cold” and “altered” tumors, various factors lead to inadequate T cell infiltration into the tumor microenvironment, resulting in poor therapeutic outcomes for immunotherapies due to the tumors’ inherent immune evasion mechanisms [8]. Consequently, researchers are actively exploring novel therapeutic strategies or combination treatments aimed at converting “cold” tumors into “hot” tumors.

The overall proportion, phenotype, and distribution of immune cells in the tumor microenvironment (TME) are important in determining the response to ICIs [9]. Cancer-associated fibroblasts (CAFs), as one of the most abundant cellular components in the TME, play a significant role in tumorigenesis and progression [10, 11]. CAFs and their downstream effectors are considered important targets for antitumor therapy [12]. With the continuous maturation of single-cell sequencing technology, it has been discovered that CAFs exhibit diverse phenotypes and significant heterogeneity. For instance, PLA2G2A^+^ CAFs can regulate tumorigenesis by modulating tumor metabolism [13], while CD63^+^ CAFs strengthen the drug tolerance of tumor cells [14]. Previous studies on melanoma have shown that FAP^+^ CAFs are enriched at the tumor edge, distributed closely to T cells, and inhibit T cell proliferation in a nitric oxide-dependent manner [15]. Recent single-cell sequencing studies on CAFs in NSCLC have revealed distinct patterns at different stages of the disease. In early-stage lung cancer, CAFs primarily express the ADH1B marker. In contrast, advanced stages of the disease are characterized by a predominance of CAFs expressing the FAP marker [16]. However, clinical trials involving FAP-targeted therapies have not yet achieved satisfactory results [17]. Therefore, it is crucial to further investigate the mechanisms by which FAP^+^ CAFs contribute to the immunotolerance of lung cancer and to develop more effective therapeutic strategies.

In the present study, we were the first to report a spatial “exclusion phenomenon” between FAP^+^ CAFs and CD8^+^ T cells in lung adenocarcinoma (LUAD). We also discovered that FAP^+^ CAFs contribute to the acidification of the TME by exporting lactate through Monocarboxylic Acid Transporter 4 (MCT4), creating a chemical barrier that hinders the infiltration and activity of CD8^+^ T cells. Further analysis revealed that FAP^+^ CAFs overexpress the long non-coding RNA LINC01711, which enhances lactate production by facilitating FGFR1-mediated phosphorylation of lactic dehydrogenase A (LDHA) at the Y10 site, promoting the formation of active LDHA tetramers. Building on these findings, we developed a small extracellular vesicle (sEV)-based delivery system to knock down LINC01711 in vivo using a synthetic siRNA construct (SC01711). In preclinical LUAD models, this approach showed promise as an adjunct to immunotherapy, significantly improving the response rate to ICIs.

## Methods

### Tissue samples

This study included tumor tissues and paired normal tissues from 56 patients with lung adenocarcinoma between 2016 and 2020 form Nanjing Medical University Affiliated Cancer Hospital (Jiangsu Cancer Hospital, Jiangsu Institute of Cancer Research, Nanjing, China). All samples were confirmed and identified by two experienced pathologists. Fresh samples were collected and embedded in paraffin to make tissue microarray (TMA). All patients have complete clinical information and follow-up data. This study was approved by the Medical Ethics Committee of Nanjing Medical University Affiliated Cancer Hospital (No.2020129). The informed consent was obtained from all subjects. The study was performed in accordance with the Declaration of Helsinki.

### Establishment of cell line-derived xenograft models

Wild-type C57BL/6 J mice (No. N000013) aged 3–4 weeks were acquired from Gem Pharmatech (China). All experiments were conducted according to the guidelines authorized by Nanjing Medical University Institutional Animal Care and Use Committee. Subcutaneous tumor bearing model and orthotopic implantation mouse model was established as we reported previously [18]. For the subcutaneous xenograft model, mice were subcutaneously injected in the 0.1 mL PBS with a cell suspension containing 2 * 10^6^ LUAD cells or LUAD cells mixed with CAF cells (at a ratio of 1:1). When a tumor was visible, measuring its volume every 3 days and calculating its volume according to the formula: volume = 0.5 * length * width^2^. For orthotopic implantation mouse model, cell suspensions of 2 * 10^6^ LUAD cells in 20 μL PBS were injected into the left lung of mice to establish the model. Then the mice were randomly divided into 2 groups, followed by treatment. In Vivo Imaging System (IVIS Lumina XR, USA) was used for tracer monitoring of the orthotopic tumor model. Tissues from the two models were used for flow cytometry experiment or immunofluorescence.

### Cell line and transfection

Murine LUAD cell line LLC1 was purchased from the cell bank of the Shanghai Institute of Cell Biology (Chinese Academy of Medical Science, Shanghai, China). The cell lines were verified by short tandem repeat (STR) profiling and screened for mycoplasma contamination. Tumor-derived CAFs were isolated from C57BL/6 J mice or human LUAD tissues while normal lung fibroblasts were isolated from C57BL/6 J mice or WT lung tissue, as previously reported [10]. To obtain FAP^+^ CAFs, we performed magnetic-activated cell sorting with anti-FAP antibody to purify the primary CAFs. In brief, the CAFs were incubated at 4° C for 30 min followed by washing. Then magnetic microbed-conjugated secondary antibody was added. FAP^–^ CAFs were collected in the flow-through of the column (Miltenyi Biotechology, Germany) and FAP^+^ CAFs in the magnetic column were then isolated by flushing out. Peripheral blood mononuclear cells (PBMCs) were isolated from blood from healthy controls as follow: Take 10 ml of fresh peripheral blood from the patient and transfer it to an EDTA anticoagulant tube and then centrifuge at 800 g for 10 min. After centrifugation, carefully take out the separation tube, which shows obvious layering. Quickly introduce the liquid above the separation layer into a new centrifuge tube and centrifuge at 300 g for 5 min. Wash the cells with 3 ml of PBS and centrifuge at 300 g for 5 min, followed by normal cultivation step and experiments. Then 4 × 10^6^ PBMC were activated with a 20 µl T Cell Activation/Expansion Kit (Miltenyi Biotec, USA) in a 1 ml T cell serum-free medium (Fcmacs, China). This medium was also supplemented with 20IL/mL IL-2 (R&D, USA). In the interests of further expansion, the density of viable cells was adjusted to 1 × 10⁶ cells/ml every 2–3 days, which was achieved by adding fresh, complete T cell serum-free medium that was supplemented with 20 IL/mL IL - 2. Cells were cultured in RPMI 1640 complete medium or Dulbecco’s modified Eagle’s medium (KeyGEN, Nanjing, China), containing 10% fetal bovine serum (FBS, Gibco, NY, USA) and 1% penicillin - streptomycin (Gibco) unless otherwise specified. The culture environment for all cells was a 5% CO₂ incubator at 37° C.

For co-culture, conditioned medium was generated by culturing CAFs (5 * 10^5^ cells, transfected or not) in a complete medium for 72 h and filtering through a 40 mm filter. Then 1 * 10^6^ PBMCs were co-cultured with mixed conditioned medium for 3 days and were collected for further analysis. To investigate the effect of lactate on CD8 positive T cells, lactate, 3-Hydroxybutyric acid (3-OBA) [19] or LDHi (GSK2837808A, Cat#HY-100681, MedChemExpress) was added into conditioned medium and followed by flow cytometry analysis.

### Fluorescence in situ hybridization (FISH) assay and Immunofluorescence

FISH assays were conducted with the lncRNA FISH Kit (GenePharma, China) according to the protocol. In brief, cells are fixed and permeabilized in PBS containing 0.5% Triton X-100 and was followed by hybridization overnight at 37° C in the dark. Cy3-probe for LINC01711 was designed by Sangon Biotech (Shanghai, Table S2). The core is stained with DAPI. Tumor tissues were stained for immunofluorescence using the TSA fluorescence double staining kit following the manufacturer’s protocols. All images were obtained with a CarlZeiss LSM710 confocal microscope (Germany) and analyzed using ImageJ. All antibody information is provided in Table S1.

### Flow cytometry

For analysis of tumor-infiltrating immune cells, tumors were removed, and single-cell suspensions were generated. Mouse-specific BD Fc Block was applied to mouse cells at 4° C for 15 min to reduce nonspecific combination. Add flow cytometry antibodies diluted according to the recommended ratio, with a required staining volume of 20 ul per well. Incubate at 4° C in the dark for half an hour, then centrifuge at 300 g for 5 min and discard the supernatant. Wash twice and conduct Flow cytometric analysis using a BD FACSAria Fusion system (BD Biosciences). For CD8-positive T cells associated analysis, the abundance of CD8-positive T cells was analyzed using flow cytometry within CD3-positive T cells, followed by analyzing CD69^+^ and GZMB^+^ CD8-positive T cells. The data were analyzed using FlowJo v10. Details on the antibodies used in flow cytometry are provided in Table S1.

### RNA interference

The Small interfering RNAs against LINC01711 or FGFR1 (si-ALDOA) and the negative control (si-NC) were designed and synthesized by Sangon Biotech (Shanghai, China). LDHA mutants were synthesized by Sangon Biotech. Lipofectamine 3000 reagent (Invitrogen, CA, USA) was used as the transfection aid reagent according to the manufacturer’s protocol. The efficiency of transfection was validated using qRT-PCR. All the interfering sequences were listed in Table S2.

### Subcellular fractionation

The subcellular expression of LINC01711 in CAFs was detected using the PARIS protein and RNA Isolation Kit (Invitrogen) according to the manufacturer’s instructions. RNA was extracted from the cytoplasmic and nuclear fractions of CAFs and then subjected to qPCR. U6 and GAPDH were used as nuclear and cytoplasmic markers, respectively.

### Glucose uptake and lactate production assay

Following transfection, 1.5 × 10^3^ cells were plated in a 96-well format and incubated for 4 days prior to usage. Cells were then processed in accordance with the manufacturer’s instructions provided in the Glucose Uptake Fluorometric Assay Kit (MAK084, Sigma-Aldrich), and glucose uptake assays were conducted. The resultant data were normalized to 10^4^ cells. For lactate production assessment, 2 × 10^6^ transfected cells were prepared as per the guidelines of the L-Lactate Assay Kit (Colorimetric) (ab65331), and the assay was performed accordingly, with results also normalized to 10^4^ cells.

### Seahorse analysis

The glycolytic activity within each cell cohort was evaluated using Seahorse XF technology with a Seahorse XF-96 Extracellular Flux Analyzer (Agilent, Santa Clara, CA, USA). In brief, cells were cultured in triplicate under conditioned media and CO^2^-free conditions for 1 h prior to calibration. After the introduction of glucose, oligomycin, and 2-DG (Sigma), the extracellular acidification rate (ECAR) for each cell cohort was recorded. The data were normalized against 10^4^ cells.

### LDHA activity and crosslinking

The LDHA activity was measured by Total LDH Assay Kit with WST-8 (P0395S, Beyotime) according to manufacturer’s protocol. Since the enzyme that mainly catalyzes the production of lactate from pyruvate is LHDA rather than LDHB, the result of this kit can roughly represent the enzyme activity of LDHA. The tetramer formation ability of LDHA, which is the active form, was detected by 0.025% glutaraldehyde crosslinking and the samples were separated in non-denaturing gels by electrophoresis.

### In vitro kinase assay

In vitro kinase assay was performed according to the steps as followed. Recombinant human LDHA variants (P01711, Solarbio) were mixed with active recombinant His tagged-FGFR1 (P09665, Solarbio) in kinase reaction buffers (HER2: 20 mM Tris (pH 7.5), 5 mM MnCl2, 0.5 mM Na3VO4, 1 mM EGTA, 2 mM DTT, 5 mM β-glycerophosphate, 0.01% CHAPS) at 30 ° C for 30 min. Terminate the reaction by soaking in a boiling water bath for 5 min. Quickly freeze the protein in liquid nitrogen and perform Western blot analysis of LDHA-Y10 phosphorylation.

### Size exclusion chromatography

Superdex 200 Increase 10/300 GL column (GE Healthcare) was used for size exclusion chromatography (gel filtration). Firstly, wash the column with distilled water and then equilibrate with PBS. Cells were lysed in lysis buffer containing 1 mM protease inhibitor mixture (Roche). Then load the protein onto the column and elute with pH 7.2 phosphate buffered saline composed of 50 mM sodium phosphate and 0.15 M NaCl. The flow rate was set to 0.5 μL/min. Fractions of 300 μL were collected, and Western blot analysis was performed using 20 μL of each fraction.

### Western blotting

Western blotting was performed according to standard protocols. In brief, cell lysates were separated by 4−12% SDS-PAGE and then transfer the protein onto a polyvinylidene fluoride (PVDF) membrane (Merck Millipore, USA). Incubate the membrane with the primary antibody overnight at 4 ° C, and then incubate with the corresponding secondary antibody. Odyssey CLx imaging system (LI-COR, USA) was used to detect and identify target proteins. All antibody information is provided in Table S1.

### RNA pull-down and RNA immunoprecipitation (RIP) assay

RNA was transcribed in vitro using the RNAmax-T7 transcription Kit (RiboBio) and biotinylated using the Pierce RNA 3’ End Desthiobiotinylation Kit (Termo Fisher Scientifc) according to the manufacturer’s instructions. RNA pull-down assays were conducted using the Pierce Magnetic RNA–Protein Pull-Down Kit (Termo Fisher Scientifc) with 50 pmol of RNA. The eluted products were subjected to western blotting and specific bands were processed by Liquid Chromatograph Mass Spectrometer (LC-MS) by Sangon Biotech (Shanghai, China). Candidate proteins are listed in Table S4. RNA Immunoprecipitation (RIP) assays were carried out with the Magna Nuclear RIP (Native) Nuclear RNA-Binding Protein Immunoprecipitation Kit (Merck Millipore) according to the manufacturer’s instructions. RNA levels were normalized to the input (10%).

### Bimolecular fluorescence complementation (BiFC)

The plasmid designed for BiFC was synthetized from Corues biotechnology (Nanjing, China). The sequence of the plasmid was shown in Table S3. The cells were transfected with FGFR1-Linker-mVenus_C and LDHA-Linker-mVenus_N, with simultaneously transfected with si-LINC01711 or not. After 3 days, the cells were stained with DAPI and were obtained image with a CarlZeiss LSM900 confocal microscope at 647 nm channel.

### Immunoprecipitation assays

Immunoprecipitation (IP) assays were performed using the Pierce Classic Magnetic IP/Co-IP Kit (Thermo Fisher Scientific) according to the manufacturer’s protocol. Cells lysates were prepared from the transfected cells. Cell lysates were incubated with Magnetic Beads. The eluates from these IPs were analyzed by western blot. To minimize background noise, primary antibodies from different biological hosts were employed to investigate the interaction of proteins.

### RT-qPCR

For reverse transcription quantitative PCR (RT-qPCR), TRIzol reagent was used to extract total RNA from tissue samples or cell specimens. 1000 ng total RNA was used for reverse transcription. RNA-to-cDNA kit was used for reverse transcription. SYBR Green Premix on a QuantStudio 6 Flex system (ABI) was applied for qPCR, with specific primers listed in Table S2.

### Design and validation of the synthetic construct for LINC01711 silencing

The synthetic construct was designed as we previously reported [20]. The primary objective of this synthetic construct is to facilitate the production and self-assembly of LINC01711-siRNA into sEVs, aiming to achieve a potential therapeutic effect. In brief, the synthetic anti-LINC01711 construct was developed by inserting the LINC01711-siRNA sequence into the pre-miR-155 scaffold downstream of the CMV promoter (Fig. S5A). Given the liver’s inherent ability to take up naked DNA plasmids and express transgenes [21, 22], the anti-LINC01711 construct absorbed by the mouse liver promotes the continuous production of pre-miRNAs in hepatocytes. These pre-miRNAs are then further processed through the endogenous RNAi mechanism to yield mature miRNA-like LINC01711-siRNAs. Subsequently, these LINC01711-siRNAs are packaged into secretory sEVs (Fig. S5A), released into the bloodstream, and transported to other tissues and organs through the body’s own circulation. To verify the effectiveness of this process, we injected plasmid into one mouse and extracted sEVs from peripheral blood after 9 h. PKH26 dye was used for sEV staining, followed by injecting into another mouse. After 18–20 h, take the main organs for section staining, qRT-PCR, and bioluminescence detection.

Orthotopic implantation mouse model was established, followed by treatment of synthetic construct. Synthetic construct for si-LINC01711 or si-NC was injected into the tail vein every 2 days. The injection of synthetic construct started 1 week after the model established.

### RNA-seq and analysis

RNA-sequence was performed using CAFs as we reported previously. Transcriptome data and clinical details were obtained from The TCGA Data Portal (https://www.cancer.gov/tcga). All 33 cancer types for which the transcriptome data were available were included in the analysis. Spearman correlations between our si-LINC01711 signature and the abundance of CD8^+^ T cells were computed using xCell, TIMER, QUANTISEQ, MCPCOUNTER, EPIC, CIBERSORT, and CIBERSORT-ABS. Motif scanning of the LINC01711 promoter region (2000bp upstream of TSS) was performed using JASPAR2022.

### Untargeted metabolomics analysis

The untargeted metabolomics analysis was performed by BestMS technologies (Shandong, China). In brief, the conditioned medium from FAP^+^ CAFs and FAP^-^ CAFs was applied for metabolomics extraction. LC/MS system was used for metabolomics analysis, which is composed of Waters Acquity I-Class PLUS ultra-high performance liquid tandem Waters Xevo G2-XS QTof high resolution mass spectrometer. The column used was purchased from Waters Acquity UPLC HSS T3 column (1.8 um 2.1 * 100 mm). The raw data collected using MassLynx V4.2 is processed by Progenesis QI software for peak extraction, peak alignment and other data processing operations, based on the Progenesis QI software online METLIN database and self-built library for identification. After normalizing the original peak area information with the total peak area, the follow-up analysis was performed. Student’s *t*-test was used to calculate the difference significance pvalue of each compound.

### U13-C Glucose stable isotope tracer analysis

The metabolic flux analysis was conducted by Shanghai Applied Protein Technology (Shanghai, China). Firstly, cells were cultured with C-13 contained medium for 12 h, followed by further processing. Then 500 μL cold extraction buffer (methanol: acetonitrile: water = 2:2:1, v/v/v) was added to each sample. Samples were sonicated for 2 min and centrifuged at 14,000 g for 5 min at 4 ° C. Supernatants were thoroughly lyophilized (FreeZone 6 Liter, Labconco, USA) and reconstituted in 50 μL of methanol-water (1:1, v/v) just prior to measurement. The MS measurement of isotopologue distribution is analyzed via a Thermo QExactive plus hybrid quadrupole–orbitrap mass spectrometer coupled to a Thermo Vanquish UPLC system. The instrument performance optimization and routine maintenance were performed every 48 h. Data processing and ion annotation based on accurate mass were performed in TraceFinder 5.0 (Thermo Fisher) and Xcalibur 4.0 (Thermo Fisher).

### Molecular docking

The receptor protein used for docking is Lactate Dehydrogenase A (LDHA), with the Uniprot ID: P00338. The three-dimensional structure of the ligand RNA was established by Xiao Lab, as detailed on their website (http://biophy.hust.edu.cn/new/). Protein preprocessing, which includes the removal of water molecules and excess ligands, as well as the addition of hydrogen atoms, was completed using PyMOL 2.4. The HDOCK SERVER (http://hdock.phys.hust.edu.cn/) was employed for the molecular docking of protein-RNA complexes. Docking Score, Confidence Score, and Ligand RMSD were utilized as the criteria for evaluating the docking results, with the model exhibiting the highest scores being selected as the optimal docking model. PyMOL was then utilized to visualize the interactions between the protein and RNA.

### Statistics analysis

Statistical analyses were conducted using SPSS 22.0 Software. Data are expressed as mean ± standard error of the mean (S.E.M.) in bar charts and line graphs. To evaluate differences in gene expression between paired tissues, a two-tailed paired Student’s t-test was utilized, whereas a two-tailed unpaired Student’s *t*-test was employed for comparisons between independent groups. One-way or two-way ANOVA was performed to analyze how the means of a quantitative variable vary with respect to one or two categorical variables, respectively. Kaplan-Meier survival curves were constructed using GraphPad Prism 9.0 Software. Statistical significance was denoted as follows: *P ≤ 0.05; **P ≤ 0.01; ***P ≤ 0.001; ****P ≤ 0.0001; n.s. indicates non-significance.

## Results

### FAP^+^ CAFs shield LUAD tumor cells from CD8^+^ T cell attacks

**Fig. 1:**
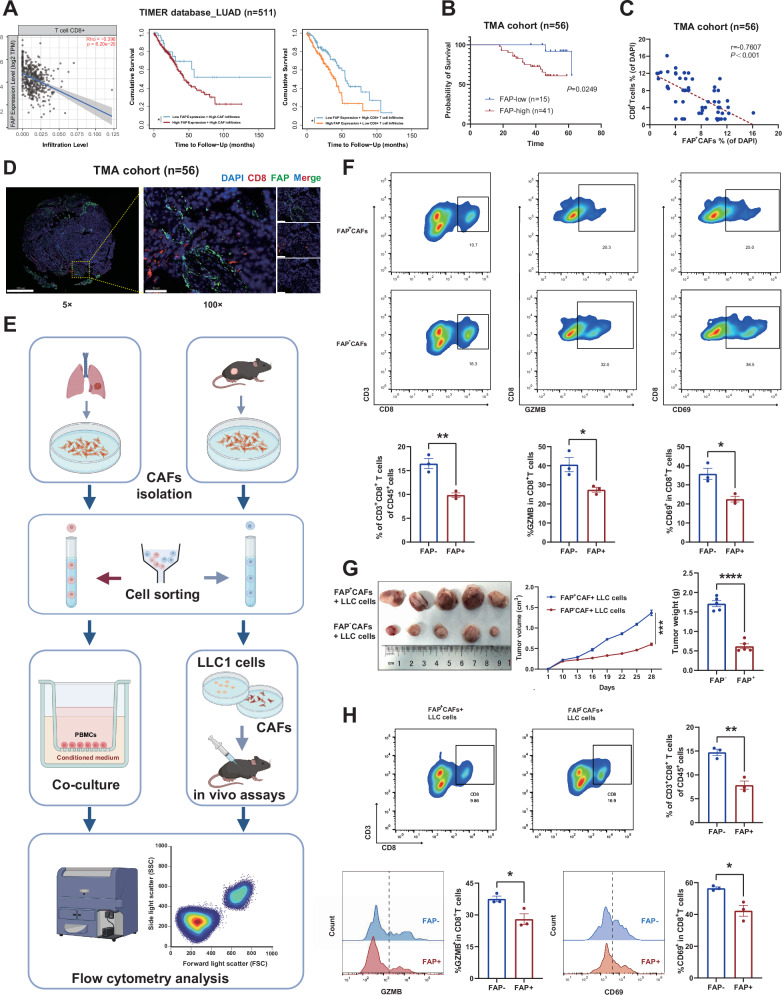
FAP^+^ CAFs shield LUAD tumor cells from CD8^+^ T cell attacks. **A** The infiltration level correlation analysis (Spearman correlation analysis) and cumulative survival analysis (Kaplan-Meier survival analysis) of FAP and CD8 in TIMER database and TCGA-LUAD database (*n* = 511). **B** Kaplan–Meier survival analysis between FAP-high (MFI of FAP ≥ 10%, *n* = 41) and FAP-low (MFI of FAP <10%, *n* = 15) group in TMA cohort. **C** Spearman correlation analysis between FAP and CD8 immunofluorescence in TMA cohort (*n* = 56). **D** Representative image of FAP and CD8 immunofluorescence in TMA cohort (*n* = 56). **E** Flow chart of in vivo and in vitro experiments. **F** Flow cytometry analysis of in vitro co-culture model (*n* = 3 biological repeats). The *P*-value was calculated by two-tailed unpaired *t*-test. **G** Representative images, tumor size, and weight of subcutaneous tumors (*n* = 5 biological repeats, the *P*-value was determined by two-way ANOVA with Tukey’s multiple comparison test or two-tailed unpaired Student’s *t*-test). **H** Flow cytometry analysis of in vivo tumor bearing model (*n* = 5 biological repeats). The *P*-value was calculated by two-tailed unpaired *t*-test. All the results were shown as mean ± S.E.M. *P ≤ 0.05, **P ≤ 0.01, ***P ≤ 0.001, ****P ≤ 0.0001.

To investigate the association between FAP^+^ CAFs and immune infiltration in LUAD, we utilized data from the TCGA-LUAD and TIMER database for further analysis. The results showed that a high abundance of FAP expression is correlated with decreased infiltration of CD8^**+**^ T cells and increased infiltration of CAFs (Fig. 1A and Fig. S1A, B). Meanwhile, the overall survival of LUAD patients with high FAP^+^ CAF infiltration is significantly lower than that of patients with low FAP^+^ CAF infiltration, with the difference becoming more pronounced when incorporating CD8^+^T cells infiltration (Fig. 1A). To further explore the relationship between FAP^+^ CAFs infiltration and CD8^**+**^ T cells infiltration in LUAD, we conducted immunofluorescence analysis using a TMA cohort (*n* = 56). Apart from similar survival analysis results (Fig. 1B), we observed that CD8^**+**^ T cells and FAP^+^ CAFs appear to be spatially mutually exclusive, with a significant negative correlation between the abundance of CD8^**+**^ T cells and FAP^+^ CAFs (Fig. 1C, D).

To further elucidate the relationship between CD8-positive T cells and FAP-positive CAFs, we designed a series of in vivo and in vitro experiments (Fig. 1E). We extracted CAFs from human LUAD tissues and from the subcutaneous tumor-bearing tissues of mice. Using flow cytometry, we differentiated FAP-positive CAFs from FAP-negative ones and confirmed their immunofluorescence identity in both primary and cultured states (Fig. S1C). In vitro, we co-cultured peripheral blood mononuclear cells (PBMCs) with conditioned medium of CAFs within a transwell system to observe the transmigration and cytotoxic capabilities of CD8^+^ T cells using flow cytometry. In the in vivo experiments, we established a subcutaneous tumor-bearing model by co-injecting tumor cells with CAFs. Once tumor formed, we analyzed the infiltration of CD8^+^ T cells by flow cytometry. Both experiments revealed that FAP^+^ CAFs, rather than FAP^-^ CAFs, significantly reduced the infiltration of CD8-positive T cells and concurrently diminished their cytotoxic capacity, evidenced by decreased expression of CD69 and Granzyme B (GZMB) (Fig. 1F–H). Moreover, the in vivo studies showed that tumors in the presence of FAP^+^ CAFs exhibited notably accelerated progression (Fig. 1G).

### The effect of FAP^+^CAFs on CD8^+^T cells depend on their dysregulated lactate production

**Fig. 2:**
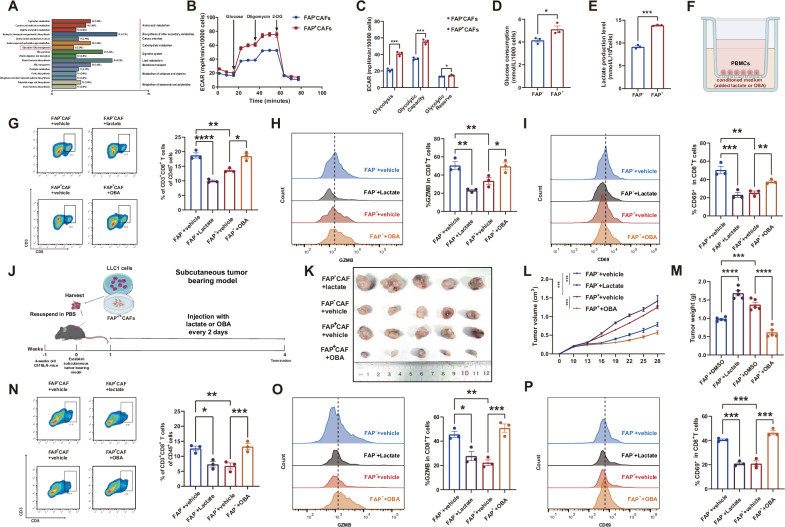
The effect of FAP^+^CAFs on CD8^+^T cells depends on their dysregulated lactate production. **A** KEGG database annotation of untargeted metabolomics analysis using FAP^+^ CAFs’ and FAP^-^ CAFs’ conditioned medium (*n* = 3 biological repeats). **B**, **C** The results of ECAR assays on FAP^+^ CAFs and FAP^-^ CAFs (*n* = 3 biological repeats). The *P*-value was calculated by two-tailed unpaired *t*-test. **D**, **E** The results of glucose uptake and lactate production measurement performed on FAP^+^ CAFs and FAP^-^ CAFs (*n* = 3 biological repeats). The *P-*value was calculated by two-tailed unpaired *t*-test. **F** Flow chart of in vitro co-culture model. **G**−**I** Flow cytometry analysis on CD8-positive T cells infiltration (**G**), GZMB^+^ CD8-positive T cells infiltration (**H**) and CD69^+^ CD8-positive T cells infiltration (**I**) in in vitro co-culture model (*n* = 3 biological repeats). The *P*-value was calculated by two-tailed unpaired *t*-test. **J** Flow chart of in vivo model. **K** Representative image of subcutaneous tumors. **L** Tumor volume of subcutaneous tumors (*n* = 5 biological repeats, the *P*-value was determined by two-way ANOVA with Tukey’s multiple comparison test). **M** Tumor weight of subcutaneous tumors (*n* = 5 biological repeats, the *P*-value was calculated by two-tailed unpaired *t*-test). **N**–**P** Flow cytometry analysis on CD8-positive T cells infiltration (**N**), GZMB^+^ CD8-positive T cells infiltration (**O**) and CD69^+^ CD8-positive T cells infiltration (**P**) in in vivo co-culture model (*n* = 5 biological repeats, the *P*-value was calculated by two-tailed unpaired *t*-test). All the results were shown as mean ± S.E.M. *P ≤ 0.05, **P ≤ 0.01, ***P ≤ 0.001, ****P ≤ 0.0001.

During the in vitro assays, we constantly noticed that the culture medium of FAP^+^ CAFs turned yellow much quicker than that of FAP^-^ CAFs, which was linked to glycolysis [23] (Fig. S1D). We also measured the expression of TGF-β and IL-10, which are immunosuppressive cytokines that CAFs typically produce, and found no significant difference (Fig. S1E). Additionally, previous studies have highlighted the detrimental impact of lactate on CD8-positive T cells. These evidences led us to hypothesize that the aerobic glycolysis efficiency of FAP^+^ CAFs may be enhanced. To verify this hypothesis, we performed untargeted metabolomics analysis and confirmed a higher concentration of glycolysis related products in the culture medium of FAP^+^ CAFs (Fig. 2A). Subsequent ECAR assays further revealed that the glycolysis efficiency of FAP^+^ CAFs is significantly higher than that of FAP^-^ CAFs (Fig. 2B, C). Experiments on glucose uptake and lactate production also yielded similar results (Fig. 2D, E). To explore whether the impact of FAP^+^CAFs on CD8^+^T cells depend on the dysregulated lactate production, we added either exogenous lactate or lactic acid receptor inhibitor 3-OBA into the conditioned medium of FAP^+^ CAFs (Fig. 2F). In the in vitro model, we found that compared with FAP^-^ CAFs + DMSO group, the infiltration and cytotoxic capacity of CD8-positive T cells was significantly reduced in FAP^-^ CAFs + lactate group. Conversely, when we added 3-OBA to the conditioned medium of FAP^+^ CAFs, both the infiltration and cytotoxic capacity of CD8-positive T cells was notably recovered (Fig. 2G–I). In our in vivo model, we injected lactate or 3-OBA into subcutaneous tumors in situ every 2 days to modulate the tumor microenvironment (Fig. 2J). Our results showed that tumor progression in the FAP^-^ CAFs + lactate group was significantly accelerated compared to the FAP^-^ CAFs + DMSO group (Fig. 2K–M), with lower infiltration and cytotoxic capacity of CD8-positive T cells. Furthermore, findings from the FAP^+^ CAFs + 3-OBA group was consistent with those observed in vitro (Fig. 2N–P).

### MCT4 and LINC01711 play a critical role in the dysregulation of lactate secretion in FAP^+^ CAFs

**Fig. 3:**
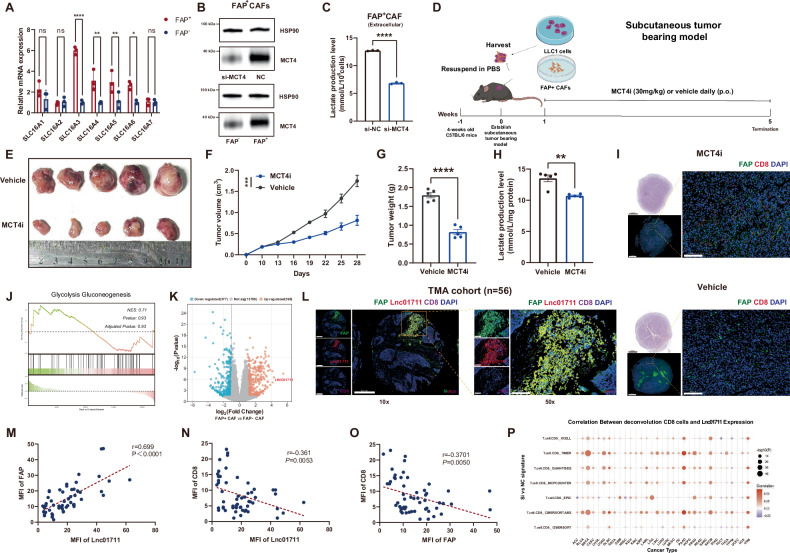
MCT4 and *LINC01711* play a critical role in lactate secretion dysregulation of FAP^+^ CAFs. **A** The result of RT-qPCR detecting the expression of the Solute Carrier family (SLC16As) in FAP^+^ CAFs (*n* = 3 biological repeats). The *P*-value was calculated by two-tailed unpaired *t*-test. **B** The result of western blotting indicating the expression of MCT4 in FAP^+^ CAFs and the efficiency of si-MCT4. **C** The extracellular lactate production level significantly decreased in FAP^+^ CAFs transfected si-MCT4 (n = 3 biological repeats). The *P*-value was calculated by two-tailed unpaired *t*-test. **D** Flow chart of in vivo co-culture model. VB124 (30 mg/kg) was orally used for MCT4 inhibition daily. **E** Representative image of subcutaneous tumors. *n* = 5 biological repeats. **F** Tumor volume of subcutaneous tumors (*n* = 5 biological repeats, the *P*-value was determined by two-way ANOVA with Tukey’s multiple comparison test). **G** Tumor weight of subcutaneous tumors (*n* = 5 biological repeats, the *P*-value was calculated by two-tailed unpaired *t*-test). **H** The lactate production level of subcutaneous tumors (*n* = 5 biological repeats, the *P*-value was calculated by two-tailed unpaired *t*-test). **I** Representative image of FAP and CD8 immunofluorescence in subcutaneous tumors. Scale bars: 2000 μm; 100 μm. **J** GSEA analysis on RNA-sequence data of FAP^+^ CAFs vs FAP^-^ CAFs. *n* = 3 biological repeats. **K** Volcano map of RNA-sequence data in FAP^+^ CAFs and FAP^-^ CAFs. *n* = 3 biological repeats. **L** Representative image of FAP and CD8 immunofluorescence and LINC01711 fluorescence in situ hybridization (FISH) in TMA cohort (*n* = 56). Scale bars: 100 μm. **M**–**O** Spearman correlation analysis between LINC01711 FISH and FAP, CD8 immunofluorescence in TMA cohort (*n* = 56). **P** The si-*LINC01711* signature was positively correlated with the infiltration of CD8^+^ T cells, as inferred by xCell, TIMER, QUANTISEQ, MCPCOUNTER, EPIC, CIBERSORT, and CIBERSORT-ABS in the TCGA cohorts. All the results were shown as mean ± S.E.M. *P ≤ 0.05, **P ≤ 0.01, ***P ≤ 0.001, ****P ≤ 0.0001.

To further understand the mechanisms behind lactate secretion dysregulation in FAP^+^ CAFs, we investigated the potential genes that involved in this process. Given that Monocarboxylic Acid Transporters (MCTs) are widely known as key lactate transporters, we firstly examined the expression of the Solute Carrier family (SLC16As), which encode MCT family proteins. As shown in Fig. 3A, B, SLC16A3, also known as MCT4, was significantly upregulated in FAP^+^ CAFs. Then we knockdown MCT4 in FAP^+^ CAFs using si-MCT4 transfection, validated by western blot (Fig. 3B). As expected, the extracellular lactate production significantly decreased in FAP^+^ CAFs transfected with si-MCT4 (Fig. 3C). Additionally, the intracellular lactate production was observed significantly increased in FAP^-^ CAFs transfected with si-MCT4 (Fig. S1H), which may be due to the accumulation of lactate. Subsequently, we conducted an in vivo experiment using VB124, an orally active, potent, and selective MCT4 inhibitor (MCT4i). Our results showed that the progression of tumor in MCT4i group was significantly slower, with smaller volume and weight (Fig. 3E–G). The lactate production level assays revealed lower lactate levels in the MCT4i group (Fig. 3H), and multiple IF staining results revealed higher infiltration level of CD8-positive T cells (Fig. 3I). Collectively, these results suggest that FAP^+^ CAF secretes lactate *via* MCT4, leading to lower infiltration of CD8-positive T cells and accelerated tumor progression.

Next, we explored the molecular mechanisms underlying the substantial lactate production by FAP^+^ CAF. We initially compared the differential gene expression between FAP^+^ CAF and FAP^-^ CAF cells by RNA-sequencing (RNA-seq), but did not identify any difference in the genes encoding key glycolytic enzymes (Fig. 3J, S1F). Interestingly, our analysis revealed that the most significantly upregulated gene in the FAP^+^ CAF group is a long non-coding RNA (lncRNA) known as LINC01711. To verify whether LINC01711 is associated with lactate abnormalities, we selected the three genes with the highest expression in the FAP + CAF group for knockdown. The results showed that only when LINC01711 was knocked down, lactate production was significantly reduced (Fig. S1G, S2A). Given the growing recognition of lncRNAs for their diverse regulatory roles in gene expression and cellular processes, we chose to focus our study on LINC01711 (Fig. 3K). Using multiplex IF staining and RNA FISH on TMAs from 56 cases, we assessed the expression of CD8, FAP, and LINC01711 in LUAD. The results revealed a significant overlap between LINC01711 and FAP expression, indicating that LINC01711 is predominantly expressed in FAP-positive cancer-associated fibroblasts (CAFs). Moreover, similar to FAP, LINC01711 showed an inverse relationship with CD8 expression, with the two exhibiting spatially distinct and mutually exclusive patterns (Fig. 3L–O).

We developed a LINC01711 signature based on the top 30 transcripts enriched of RNA-seq analysis on LINC01711-knockdown FAP^+^ CAFs. We observed that the si-LINC01711 signature was positively correlated with the infiltration of CD8^+^ T cells, as inferred by xCell, TIMER, QUANTISEQ, MCPCOUNTER, EPIC, CIBERSORT, and CIBERSORT-ABS within the TCGA cohorts (Fig. 3P). We then validated the expression of LINC01711 in normal fibroblast cells, LUAD cell lines and CAFs by RT-qPCR, as well as TMA cohort and TCGA-LUAD cohort validation. The result showed that LINC01711 is upregulated in tumor tissue and is specifically overexpressed in FAP^+^ CAFs (Fig. S2B–D). As reported by the GEPIA database (http://gepia.cancer-pku.cn/), LDHA expression was elevated in LUAD patients and correlated with poor prognosis (Fig. S2E, F). Additionally, GSEA analysis revealed that LINC01711 knockdown showed no significant difference in glycolysis pathway (Fig. S2G). Coding potential assessment using CPAT, PRIDE reprocessing 2.0, PhyloCSF score indicated that a low coding score of LINC01711 which was consistent with the characteristics of lncRNAs (Fig. S2H). Furthermore, to investigate the upstream regulation of LINC01711, we also performed an integrative computational analysis combining public TCGA-LUAD transcriptomic data, motif scanning using the JASPAR database. Intersecting the two sets yielded two transcription factors (MEIS3 and TWIST1) that were both co-expressed with LINC01711 and predicted to bind its promoter (Fig. S2I, K). RT-qPCR also confirmed that the abundance of MEIS3 and TWIST1 in FAP^+^CAFs was higher than that in FAP^-^ CAFs (Fig. S2L).

### LINC01711 enhances aerobic glycolysis in FAP^+^ CAFs by binding to LDHA

**Fig. 4:**
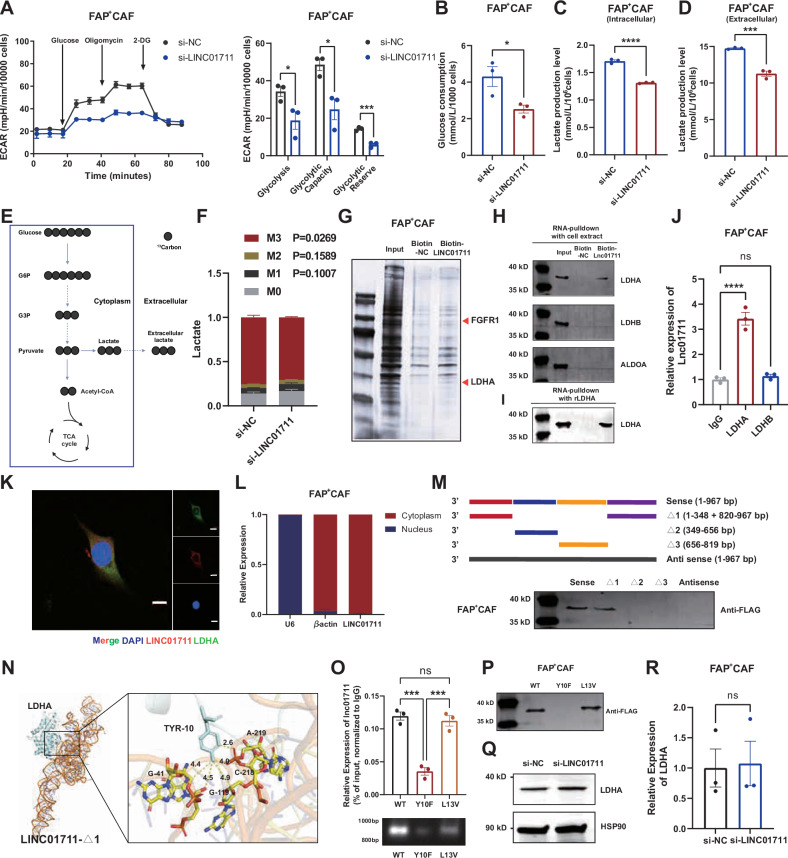
*LINC01711* enhances aerobic glycolysis *via* binding to LDHA. **A** The results of ECAR assays performed in FAP^+^ CAFs transfected with si-LINC01711 or si-NC (*n* = 3 biological repeats). The *P*-value was calculated by two-tailed unpaired *t*-test. **B**–**D** The results of glucose uptake, intracellular lactate production and extracellular lactate production measurement performed in FAP^+^ CAFs transfected with si-LINC01711 or si-NC (*n* = 3 biological repeats). The *P*-value was calculated by two-tailed unpaired *t*-test. **E** Flow chart of [U13 C] Glucose stable isotope tracer analysis. **F** [U13 C] Glucose stable isotope tracer analysis was performed in FAP^+^ CAFs transfected with si-LINC01711 or si-NC. The lactate was shown (*n* = 3 biological repeats). The *P*-value was calculated by two-tailed unpaired *t*-test. **G** Silver SDS-PAGE (sodium dodecyl sulfate-polyacrylamide gel electrophoresis) image revealing proteins immunoprecipitated by LINC01711 and its antisense RNA in FAP^+^ CAFs. **H** Western blotting validated the interaction between LINC01711 and LDHA. **I** RNA-pulldown assay was performed using biotin-LINC01711 and recombinant LDHA, followed by western blotting validation. **J** RIP (RNA immunoprecipitation) assay followed by RT-qPCR analysis confirmed that LINC01711 bound to LDHA, rather than LDHB (*n* = 3 biological repeats). The *P*-value was calculated by two-tailed unpaired *t*-test. **K** Dual RNA-FISH (fluorescence in situ hybridization) and immunofluorescence assay showing the colocalization of LINC01711 and LDHA in FAP^+^ CAFs. Scale bars: 10 μm. **L** RT-qPCR detection of LINC01711 expression in the cytoplasmic and nuclear fractions of FAP^+^ CAFs. **M** Immunoblot detection of LDHA protein in FAP^+^ CAFs by searching for biotinylated RNA or its antisense sequence of LINC01711 isoform transcribed in vitro. **N** Molecular docking predicted 3D structure of the LDHA-LINC01711-Δ1 complex. **O** RIP (RNA immunoprecipitation) assay followed by RT-qPCR analysis confirmed the interaction between LDHA-mutant and LINC01711 (*n* = 3 biological repeats). The *P*-value was calculated by two-tailed unpaired *t*-test. **P** Western blotting validated the interaction between the interaction between LDHA-mutant and LINC01711. **Q** Western blotting confirmed that altering LINC01711 expression would not affect LDHA expression. **R** RT-qPCR confirmed that altering LINC01711 expression would not affect LDHA expression (*n* = 3 biological repeats). The *P*-value was calculated by two-tailed unpaired *t*-test. All the results were shown as mean ± S.E.M. *P ≤ 0.05, **P ≤ 0.01, ***P ≤ 0.001, ****P ≤ 0.0001.

Given the evidences above, we then investigated whether LINC01711influrences glycolysis of FAP^+^ CAFs. We knockdown LINC01711 in FAP^+^ CAFs using siRNA and performed ECAR assays, glucose uptake assays and lactate production assays. We found that the glycolytic capacity of FAP^+^ CAFs significantly decreased when LINC01711 was knockdown (Fig. 4A–D). Additionally, U13-C Glucose stable isotope tracer analysis confirmed that LINC01711 knockdown reduced the lactate production in FAP^+^ CAFs (Fig. 4E, F).

We further explored the specific mechanisms by which LINC01711 regulates glycolysis in FAP^+^ CAFs. Since no differences were detected in the genes encoding key glycolytic enzymes, we excluded the probability that LINC01711 influences glycolysis by modulating mRNA expression. According to extensive reports, lncRNAs exert their biological effects by binding to proteins. Thus, we conducted RNA-pulldown assay to uncover the mechanism underlying the role of LINC01711 and subjected the products to mass spectrometry analysis to identify the potential LINC1711-binding proteins. The silver staining results showed that several bands of proteins potentially combined with LINC01711 were distributed in the 35-40 kDa and ~100 kDa regions (Fig. 4G). According to the unique peptides and western blot validation, we confirmed that LINC01711 could bind to LDHA (Fig. 4H), which is a key enzyme in glycolysis, catalyzing the production of lactic acid from pyruvic acid, rather than LDHB or ALDOA. We also performed RNA-pulldown assay with biotin-LINC01711 and recombinant LDHA, as well as RIP assays for validation of this interact (Fig. 4I, J).

Furthermore, the colocalization of LINC01711 and LDHA was also observed by dual RNA-FISH and immunofluorescence assay, confirming their spatial interaction (Fig. 4K). RT-qPCR analysis of cytoplasmic and nuclear fractions indicated that LINC01711 is predominantly localized in the cytoplasm (Fig. 4L). To clarify the specific sequence of LINC01711 that binds to LDHA, we constructed a series of LINC01711 deletion mutants based on its stem-loop structures (Fig. 4M and S2L). The results showed that deletion of the RNA fragment ranging from 1-348 + 820-967 bp (LINC01711-Δ1) significantly decreased the ability of binding to LDHA, which revealed that this region was critical for the interaction of LINC01711 with LDHA. Finally, we performed molecular docking to predict the interaction between LINC01711-Δ1 and LDHA. The Docking score is -323.43, and the Y10 site of LDHA interacts most frequently with LINC01711-Δ1, with 5 sites of LINC01711-Δ1 interacting with the Y10 amino acid site of LDHA (Fig. 4N). Then we constructed two LDHA mutants, LDHA Y10F and LDHA L13V, according to molecular docking results, followed by RNA-pulldown and RIP assays. Compared to WT LDHA group, LDHA Y10F mutant, rather than L13V mutant, showed a significantly weakened interaction with LINC01711, indicating the critical role of Y10 site (Fig. 4O, P). Together, both the 1-348 + 820-967 bp of LINC01711 and Y10 site of LDHA are essential for their interaction, which enhances aerobic glycolysis in FAP^+^ CAFs.

### LINC01711 modulates LDHA phosphorylation and tetramer formation *via* FGFR1

**Fig. 5:**
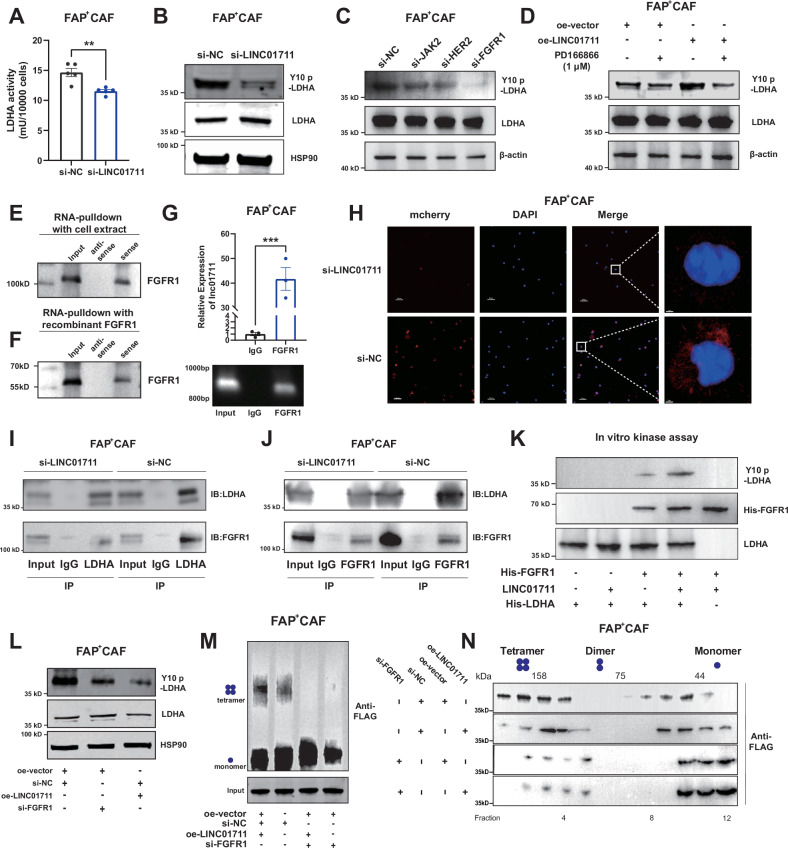
*LINC01711* modulates the phosphorylation and tetramer formation of LDHA by FGFR1. **A** LDHA activity detection revealed that LINC01711 knockdown inhibited LDHA activity (*n* = 5 biological repeats). The *P*-value was calculated by two-tailed unpaired *t-*test. **B** Western blot revealed that phosphorylation level of LDHA significantly decreased when LINC01711 was knockdown. **C** The results of western blot indicated the level of phosphorylation at the Y10 site of LDHA when FGFR1, Her2 or JAK was knocked down. **D** Western blot detected the level of phosphorylation at the Y10 site of LDHA in FAP^+^ CAFs treated with PD166866, oe-LINC01711, and both. **E**, **F** RNA-pulldown assay was performed using biotin-LINC01711 and endogenous FGFR1, or biotin-LINC01711 and recombinant LDHA, followed by western blotting validation. **G** RIP (RNA immunoprecipitation) assay followed by RT-qPCR analysis confirmed that LINC01711 bound to FGFR1 (*n* = 3 biological repeats). The *P*-value was calculated by two-tailed unpaired *t*-test. **H** BiFC (Bimolecular fluorescence complementation) experiment revealed LINC01711 could modulates the phosphorylation by FGFR1. **I**, **J** Immunoprecipitation experiment revealed that LINC01711 could promotes the interaction between FGFR1 and LDHA. **K** The in vitro kinase assay was conducted using recombinant His-tagged LDHA and His-tagged FGFR1. The result showed that LINC01711 could significantly promote FGFR1 mediated phosphorylation of LDHA. **L** Western blot, indicated that overexpression of LINC01711 could upregulated LDHA phosphorylation and the process depends on FGFR1. **M** Crosslinking followed by western blot revealed that LINC01711 could promote the tetramer formation of LDHA, which was dependent on the participant of FGFR1. **N** The result of size exclusion chromatography followed by western blot was consistent with crosslinking results. All the results were shown as mean ± S.E.M. *P ≤ 0.05, **P ≤ 0.01, ***P ≤ 0.001, ****P ≤ 0.0001.

We investigated how LINC01711 influences the biological functions of LDHA. We initially validated whether LINC01711 affects LDHA expression. After reducing LINC01711 expression in FAP^+^ CAFs and conducting RT-qPCR, TCGA-LUAD cohort analysis, and western blot experiments, we observed no significant changes in LDHA expression (Fig. 4Q, R and Fig. S3A). The Y10 amino acid site of LHDA was previously reported to be a key phosphorylation site [24], which modulates the enzyme activity and tetramer formation of LDHA. Following LINC01711 knockdown in FAP^+^ CAFs, we assessed the activity and phosphorylation level of LDHA, both of which remarkably decreased (Fig. 5A, B).

Previous studies have demonstrated that several kinases promote the phosphorylation of the FGFR1 Y10 site in various cell lines, such as JAK, HER2, and FGFR1 [24–26]. We synthesized siRNA for each kinase and knocked them down individually in FAP^+^CAF cells (Fig. S3B–D). Knocking down FGFR1 notably reduced the phosphorylation level of LDHA, particularly at the Y10 site (Fig. 5C). Using the FGFR1 inhibitor, PD1666866, further confirmed these similar findings: LDHA phosphorylation significantly decreased in the FGFR1 inhibitor group, especially when LINC01711 was overexpressed (Fig. 5D). Upon revisiting our silver staining mass spectrometry data, we identified FGFR1 as a potential binding partner (Fig. 4G). To clarify the interaction of FGFR1 and LINC01711, we performed RNA-pulldown assays using cell extracts and recombinant FGFR1 (Fig. 5E, F). The result showed that LINC01711 binds to FGFR1, followed by further validation in RIP assays (Fig. 5G). Additionally, RT-qPCR and TCGA-LUAD cohort data analysis showed that changes in LINC01711 expression do not affect FGFR1 expression (Fig. S3E–G).

Given the evidence that FGFR1 promotes LDHA phosphorylation at the Y10 site, we conducted the bimolecular fluorescence complementation (BiFC) and IP experiments. In LINC01711-kncokdown FAP^+^ CAFs, the interaction between FGFR1 and LDHA was significantly reduced, highlighting the critical role of LINC01711 in LDHA phosphorylation (Fig. 5H–J). Furthermore, we applied recombinant His-tagged LDHA and His-tagged FGFR1 to conducted in vitro kinase assay. The result showed that LINC01711 significantly enhances FGFR1-mediated LDHA phosphorylation (Fig. 5K). Western blot analysis directly assessed the LDHA phosphorylation level at the Y10 site. Results indicated that overexpression of LINC01711 increased LDHA phosphorylation in FGFR1-dependent manner (Fig. 5L). Previous studies reported that the Y10 site could affect LDHA tetramer formation, which is the active form of the enzyme. Therefore, we evaluated the tetramer formation ability of LDHA using crosslinking and size-exclusion chromatography (SEC). Consistent with our previous observations, the overexpression of LINC01711 enhanced LDHA tetramer formation contingent on FGFR1 presence (Fig. 5M, N).

### LINC01711 enhances aerobic glycolysis in FAP^+^ CAFs and shields against CD8^+^ T cell attacks *via* the FGFR1/LDHA complex

**Fig. 6:**
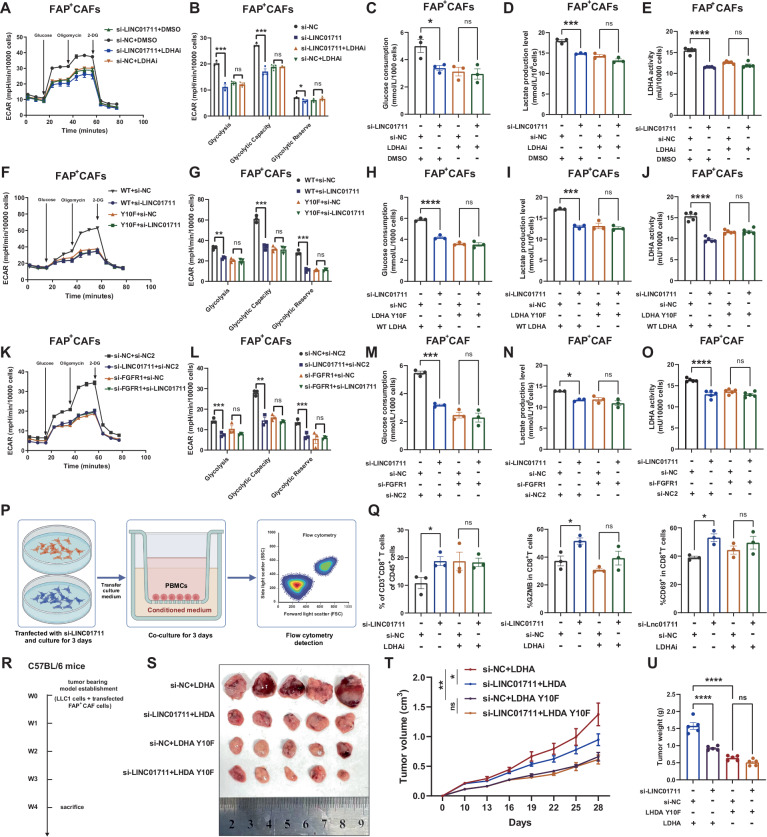
*LINC01711* facilitates aerobic glycolysis in FAP^+^ CAFs and shields against CD8^+^ T cell attacks *via* the FGFR1/LDHA complex. **A**–**E** ECAR assay, glucose uptake assay, lactate production assay and LDHA activity assay were performed in FAP+ CAFs transfected with si-LINC01711 or si-NC, treated with LDH inhibitor or not (*n* = 3 biological repeats). The *P*-value was calculated by two-tailed unpaired *t*-test. **F**–**J**. ECAR assay, glucose uptake assay, lactate production assay and LDHA activity assay were performed in FAP^+^ CAFs transfected with si-LINC01711 or si-NC, co-transfected with LDHA WT or LDHA mutant (*n* = 3 biological repeats). The *P*-value was calculated by two-tailed unpaired *t*-test. **K**–**O** ECAR assay, glucose uptake assay, lactate production assay and LDHA activity assay were performed in FAP^+^ CAFs transfected with si-LINC01711 or si-NC, co-transfected with si-FGFR1 or si-NC (*n* = 3 biological repeats). The *P*-value was calculated by two-tailed unpaired *t*-test. **P** Flow chart of in vitro co-culture model. **Q** Flow cytometry analysis on CD8-positive T cells infiltration, GZMB^+^ CD8-positive T cells infiltration and CD69^+^ CD8-positive T cells infiltration in in vitro co-culture model. **R** Flow chart of in vivo model. **S** Representative image of subcutaneous tumors. *n* = 5 biological repeats. **T** Tumor volume of subcutaneous tumors (*n* = 5 biological repeats, the *P*-value was determined by two-way ANOVA with Tukey’s multiple comparison test). **U** Tumor weight of subcutaneous tumors (*n* = 5 biological repeats, the *P*-value was calculated by two-tailed unpaired *t*-test). All the results were shown as mean ± S.E.M. *P ≤ 0.05, **P ≤ 0.01, ***P ≤ 0.001, ****P ≤ 0.0001.

Firstly, we demonstrated that FGFR1 knockdown, LDHA inhibition or LHDA mutant transfection does not affect the LINC01711 expression (Fig. S3H–K). To further explore whether LINC01711 promotes aerobic glycolysis in FAP^+^ CAFs through the FGFR1/LDHA complex, we conducted a series of validation experiments. Our results demonstrated that inhibiting LDHA activity prevented LINC01711 from promoting aerobic glycolysis in FAP^+^ CAFs. This suggests LINC01711’s effect on glycolysis depends on LDHA (Fig. 6A–E). Additionally, when we transfected FAP^+^ CAFs with WT LDHA or the LDHA Y10F mutant plasmid (Due to the plasmid transfection resulting in LDHA levels several-fold higher than the endogenous levels, making endogenous LDHA negligible, Fig. S3L), LINC01711 knockdown could only affect aerobic glycolysis in the WT LDHA group instead of LDHA Y10F group (Fig. 6F–J). Furthermore, LINC01711 knockdown also would not promote aerobic glycolysis in FAP^+^ CAFs when FGFR1 was knocked down (Fig. 6K–O). To assess whether LINC01711 could shield against CD8^+^ T cell attacks, we performed co-culture experiments (Fig. 6P). As shown in Fig. 6Q, LINC01711 knockdown significantly shielded FAP^+^ CAFs against CD8^+^ T cell attacks. However, when LDHA activity was inhibited, knocking down LINC01711 no longer provided this protective effect. Additionally, in vivo model revealed that the protective effect of knocking down LINC01711 was dependent on the regulation of LDHA phosphorylation (Fig. 6R–U). These results indicate that LINC01711 shield FAP^+^ CAFs against CD8^+^ T cell attacks, which is dependent on LDHA activity regulation.

### Delivery of in vivo self-assembled LINC01711-siRNA encapsulated in small extracellular vesicles reverses FAP^+^ CAFs-mediated immune evasion to enhance LUAD immunotherapy

Based on the evidence above, we explored whether knocking down LINC01711 could reverse FAP^+^ CAFs mediated immune evasion, which may provide novel insights for targeting these cells. We initially compared the effects of LINC01711 knockdown with LDHA inhibitors on the glycolytic efficiency of FAP^+^ CAFs. Reducing LINC01711 expression mirrored the effects of LDHA inhibitors in decreasing glycolysis in FAP^+^ CAFs (Fig. S4A–E). Importantly, knocking down LINC01711 did not affect PBMCs glycolysis (Fig. S4F), prompting us to focus on its potential clinical applications.

Our team previously developed a novel siRNA delivery system using synthetic biology strategies. By injecting specific synthetic constructs into mice *via* tail veins, hepatocytes were engineered to self-assemble, synthesize, and secrete small extracellular vesicles (sEVs) encapsulating siRNA. These sEVs then utilize the circulatory system to target tissues or cells, inducing gene silencing effects [20, 27–29]. In this experiment, the si-LINC01711 were inserted downstream of the CMV promoter into the pre-miR-155 backbone (Fig. S5A). Our studies have demonstrated that the pre-miR-155 backbone is capable of stably carrying and expressing the embedded siRNA, and following self-assembly and secretion by the liver, the siRNA can efficiently accumulate in pulmonary tissues [27]. We organized each component in the form of a naked DNA plasmid and injected it into mice *via* tail vein. Owing to the liver’s inherent ability to uptake naked DNA vectors and express transgenes [21, 22], the anti-LINC01711 synthetic constructs absorbed by the mouse liver promoted the continuous production of pre-miRNAs in hepatocytes. These pre-miRNAs were further processed to generate mature miRNA-like anti-LINC01711 siRNAs, which were subsequently spontaneously packaged into secretory sEVs (Fig. S5A) and efficiently transported to lung tissues. Specifically, we injected 5 mg/kg of the synthetic construct into tumor bearing or normal C57BL/6 J mice *via* tail vein. Nine hours post-injection, we collected sEVs from the blood, labeled them with PKH26, and reinjected them into another batch of C57BL/6 J mice. Eighteen to twenty hours later, we performed bioluminescence imaging, tissue sectioning, and RT-qPCR validation on the mice. The results showed that the synthetic construct delivery system effectively knock down LINC01711 in vivo (Fig. S5B, C). RT-qPCR, bioluminescence imaging and subsequent tissue scans further indicated successful delivery of si-LINC01711 to the lung (Fig. S5D–G). As anti-LINC01711 synthetic constructs was processed in hepatocytes, we performed liver H & E staining of treated mice and found that there is no obvious hepatotoxicity (Fig. S5H). Additionally, RNA-seq revealed that no significant off-target effect was raised by anti-LINC01711 synthetic constructs treatment (Fig. S5I).

**Fig. 7:**
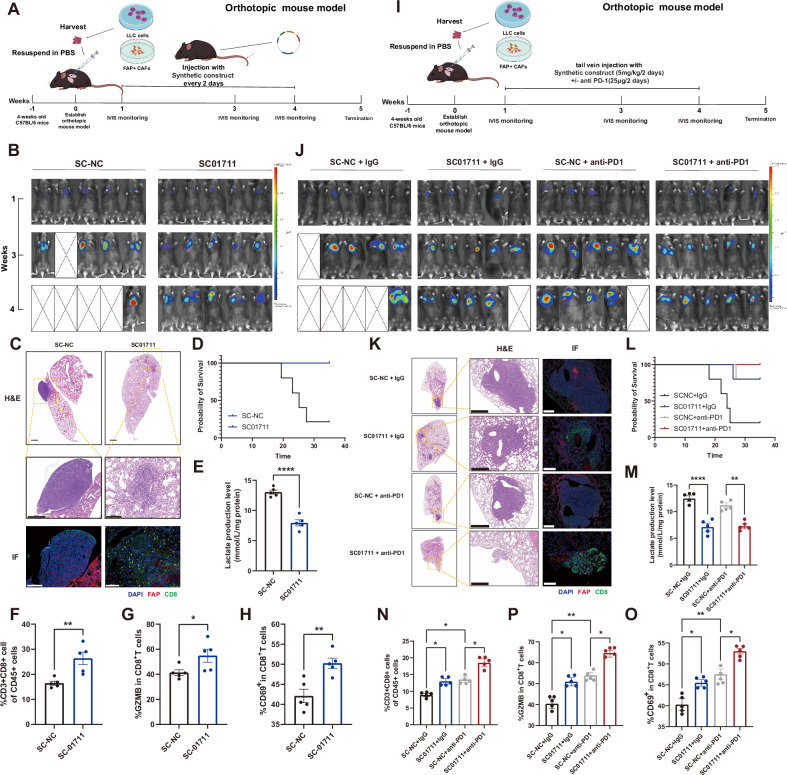
Targeting *LINC01711* in vivo using sEVs reverses FAP^+^ CAFs mediated immune evasion to enhance immunotherapy in lung adenocarcinoma. **A** Flow chart of in vivo experiments using synthetic construct carried si-LINC01711 (SC01711). **B** Representative images of IVIS detection in the orthotopic mouse model. **C** The H&E staining and IF of serial sections from the first orthotopic mouse model. Scale bars: 500 μm or 100 μm. **D** The mice OS were measured. **E** The lactate levels in mice blood were measured (*n* = 5 biological repeats). The *P*-value was calculated by two-tailed unpaired *t*-test. **F**–**H** Flow cytometry analysis on CD8-positive T cells infiltration, GZMB^+^ CD8-positive T cells infiltration and CD69^+^ CD8-positive T cells infiltration in orthotopic mouse model (*n* = 5 biological repeats). The *P*-value was calculated by two-tailed unpaired *t*-test. **I** Flow chart of in vivo experiments using synthetic construct carried si-LINC01711 (SC01711) and an anti–PD- 1 antibody. **J** Representative images of IVIS detection in the second orthotopic mouse model. **K** The H&E staining and IF of serial sections from the second orthotopic mouse model. Scale bars: 500 μm or 100 μm. **L** The mice OS were measured. **M** The lactate levels in mice blood were measured (*n* = 5 biological repeats). The *P*-value was calculated by two-tailed unpaired *t*-test. **N**–**P** Flow cytometry analysis on CD8-positive T cells infiltration, GZMB^+^ CD8-positive T cells infiltration and CD69^+^ CD8-positive T cells infiltration in the second orthotopic mouse model (*n* = 5 biological repeats). The *P*-value was calculated by two-tailed unpaired *t*-test. All the results were shown as mean ± S.E.M. *P ≤ 0.05, **P ≤ 0.01, ***P ≤ 0.001, ****P ≤ 0.0001.

Then we established orthotopic lung tumor mouse model for assessment. The lungs of C57BL/6 J mice were orthotopically implanted with LLC1-luc cells and FAP^+^CAFs at a ratio of 1:1. After 1 week, tumor formation was confirmed using an in vivo imaging system (IVIS) and then treated with synthetic construct si-LINC01711 (SC01711, 5 mg/kg each time) or synthetic construct si-NC (SC-NC) every 2 days (Fig. 7A). The mice in the SC01711 group exhibited significantly decelerated tumor progression, evidenced by lower total flux in IVIS (Fig. 7B). The results of immunofluorescence indicated lower infiltration the of CD8-positive T cells (Fig. 7C). Moreover, the mice in the SC01711 group had higher body weight and extended survival compared with the SC-NC group (Fig. S5J, K). Additionally, the lactate levels of tumors in SC01711-treated mice were notably reduced (Fig. 7E). Moreover, the results of flow cytometry analysis indicated that tumors of SC01711-treated mice were with lower infiltration and cytotoxic capacity of CD8-positive T cells (Fig. 7F–H). Furthermore, we treated mice with orthotopic lung tumors using SC01711 alongside an anti-PD-1 antibody. We then evaluated how the SC01711 treatment impacted the effectiveness of the anti-PD-1 immunotherapy (Fig. 7I). The combination treatment consisting of SC01711, and anti-PD-1 antibody exerted more potent tumor- suppressing effects than either treatment alone (Fig. 7J–L and S5L, M). The lactate production level has also been assessed (Fig. 7M). The combination treatment also increased infiltration and cytotoxic capacity of CD8-positive T cells compared with the effect of either single treatment (Fig. 7N, O, P), consistent with immunofluorescence results (Fig. 7K). These findings suggested that combination therapy consisting of SC01711 and anti-PD-1 antibodies showed potential benefit for tumor immunotherapy.

## Discussion

Although ICIs targeting PD-1 or CTLA-4 have achieved remarkable success in lung cancer treatment recently, their overall efficacy remains limited. Only a subset of patients exhibit positive responses and derive substantial benefits from ICIs [4]. Moreover, most patients who initially respond to immune checkpoint inhibitors eventually develop acquired resistance to ICIs. The low response rate to ICIs is likely associated with the prevalence of the “cold” tumor in many patients, characterized by reduced infiltration of CD8^+^ T cells [7]. In this study, we identified that FAP positivity in late-stage LUAD may be one of the potential causes leading to the formation of “cold” tumors. Our findings revealed the presence of a lactate-rich chemical barrier surrounding FAP^+^ CAFs. Further mechanistic exploration revealed that FAP^+^ CAFs exhibit high expression of LINC01711, which promotes FGFR1-mediated phosphorylation of LDHA and promotes the formation of active tetramers. This process drives increased lactate production by FAP^+^ CAFs, which is then transported into the tumor microenvironment via MCT4. To address this, we developed an sEV-based system for in vivo knockdown of LINC01711 and demonstrated its ability to sensitize LUAD to immunotherapy in a mouse model, highlighting its potential translational value.

Although anti-FAP radioimmunoconjugates achieved success in diagnostic application, the therapeutic effect of FAP-targeted therapies remains unsatisfactory [30]. Current strategies targeting FAP^+^ CAFs include inhibiting FAP’s proteolytic activity using small molecules or antibodies, vaccination strategies directed against FAP, and CAR-T cell therapies [31]. Previous efforts to target FAP^+^ CAFs in malignant tumors have been extensive, with numerous clinical trials conducted. However, many phase I and phase II clinical trials resulted in failure due to low affinity, poor specificity, and adverse side effects [17, 32–35]. For instance, Sibrotuzumab, the first humanized monoclonal antibody against FAP which was demonstrated high affinity for FAP^+^ CAFs in vitro yet failed to prove its efficacy in clinical trials [36]. Additionally, Talabostat, a FAP enzyme inhibitor, also showed no significant therapeutic effect in clinical trials involving patients with non-small cell lung cancer [32]. While various FAP-targeting small molecules or antibodies were demonstrated safe and tolerable, the therapeutic effect is often limited to preclinical models. Regarding the CAR-T cell therapies, studies demonstrated FAP CAR-T cells reduced tumor growth in murine models without notable toxicity and weight loss [37, 38]. However, toxic responses such as cachexia and lethal osteotoxicity, limited sources of cells and the immunosuppressive TME remain a challenge for CAR-T cell therapies [39]. Further clinical trials seem to be essential to confirm the safety and efficacy of CAR-T cell therapy in the clinical setting. The limited efficacy of small molecules, antibodies and CAR-T cell therapy could be caused by TME-associated factors. Our study indicated that a lactate chemical barrier surrounding FAP^+^ CAFs could cause the reduced infiltration of CD8^+^ T cells, which could be one of the reasons for the failure of clinical trials. Notably, the application of SC01711 was proved to break the lactate chemical barrier surrounding FAP^+^ CAFs in preclinical models without significant hepatotoxicity, showing great translational potential in FAP-target therapies.

Lactate has been shown to suppress the proliferation of both human cytotoxic T lymphocytes (CTLs) and CD4^+^ T cells [40]. This suppression can be up to 95% for CTLs and leads to a significant decrease in cytotoxic activity, which can be reversed by a recovery period in lactic acid-free medium. High levels of lactate in the tumor environment can block lactic acid export in T cells, thereby disturbing their metabolism and function. This suggests that lactate has a regulatory effect on invading immune cells, contributing to tumor-induced immunosuppression. Furthermore, lactate exposure induces reductive stress in T cells, shifting the NAD + /NADH redox state and depleting the reactions of glyceraldehyde 3-phosphate dehydrogenase (GAPDH) and phosphogluconate dehydrogenase (PGDH) of NAD + . This deprivation of glucose-derived serine is necessary for effector T cell proliferation [41]. The immunomodulatory control of lactate on CD8 + T cells was revealed dependent on the pH changes induced by lactic acid rather than lactate itself [42]. The acidic form of lactate, derived from tumor cells or other cells, inhibits CD8 + T cell cytotoxicity [43]. The related molecular mechanism includes metabolism regulation, altering PD-1 expression and affecting granule exocytosis [43–45]. Conversely, exogenous sodium lactate, rather than tumor-derived lactic acid, was reported to play an immune-protective role in antitumor immunity. Feng et al. revealed that sodium lactate increases intracellular lactate concentration without interfering with tumor acidity, thereby enhancing the stemness of CD8^+^ T cells and boosting anti-tumor immunity [46]. Additionally, Barbieri et al. confirmed that exposure to lactate in a pH-neutral environment can enhance the stemness of CD8^+^ T cells [47]. In our study, we revealed that FAP^+^CAFs upregulate lactate in TME by upregulating LDHA enzyme activity, leading to a decrease in CD8^+^T cell cytotoxicity. The regulatory effect of lactate derived from FAP^+^ CAFs on CD8^+^ T cells is similar to lactate derived from tumors, depending on its acidic form. Further researches are needed to reveal the specific bidirectional effects and molecular mechanisms of lactate on CD8^+^ T cells. In addition to these traditional views, several recent studies reported that lactate may also have positive effects on CD8^+^ T cells.

Although knockdown of lncRNA using antisense oligonucleotides (ASOs) is an ideal method for lncRNAs in the nucleus, there is currently a lack of ideal methods for inhibiting lncRNAs in the cytoplasm. We have developed a method to reduce the presence of cytoplasmic lncRNAs in living organisms by using an exosome delivery system. Both exosomes and lipid nanoparticles (LNPs) are widely used for delivering therapeutic agents, and each comes with distinct advantages and disadvantages. Exosomes, which are naturally derived, offer lower risks of immune reaction and toxicity, making them suitable for repeated use and effective at crossing the blood-brain barrier [48]. However, their complexity makes large-scale production difficult. On the other hand, LNPs can be mass-produced efficiently with high encapsulation rates and customizable features. However, they may provoke immune responses and have potential toxicity issues [49]. Considering these factors, we opted for exosomes as our delivery method to reduce the risk of side effects associated with treatment.

In summary, we identified the presence of a lactate chemical barrier surrounding FAP^+^ CAFs, which may contribute to the formation of “cold” tumors in patients with FAP-positive LUAD. We also discovered that FAP^+^ CAFs specifically overexpress LINC01711, which promotes LDHA phosphorylation and active tetramers formation, ultimately leading to increased lactate production. Furthermore, we demonstrated that using sEVs as a delivery system to knock down LINC01711 could successfully enhance the infiltration of CD8^+^ T cells within the tumor, providing a novel approach to sensitize lung adenocarcinoma to immunotherapy.

## Supplementary information

Figure S1

Figure S2

Figure S3

Figure S4

Figure S5

Table S1-S4 and supplement figure legends

Full and uncropped western blots.

## Data Availability

The RNA-seq data supporting the findings of this study was uploaded to GEO database (GSE301076). The LC-MS data was presented in Table S4.
