# Supplementary figures and images for: Targeting LINC01711 in FAP+ cancer-associated fibroblasts overcomes lactate-mediated immunosuppression and enhances anti-PD-1 efficacy in lung adenocarcinoma
Source: Cell Death Dis. 2025 Aug 25;16(1):642. doi: 10.1038/s41419-025-07974-6 (PMC12379239; doi:10.1038/s41419-025-07974-6)

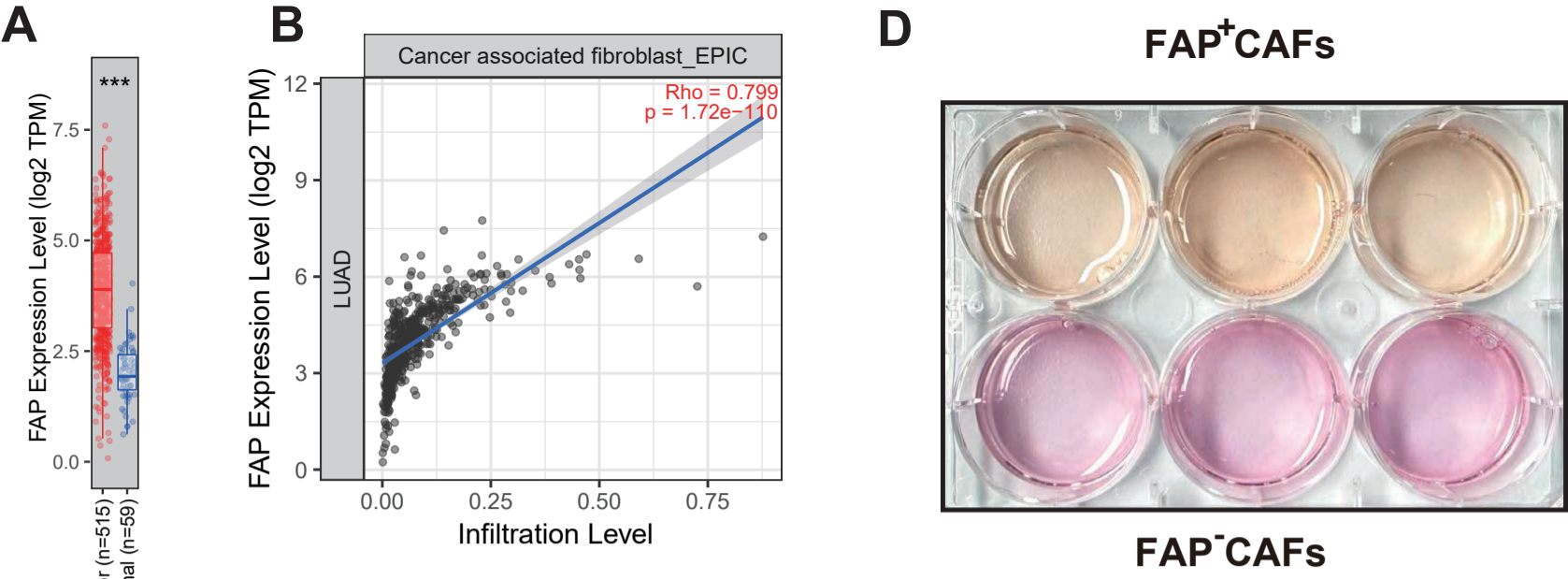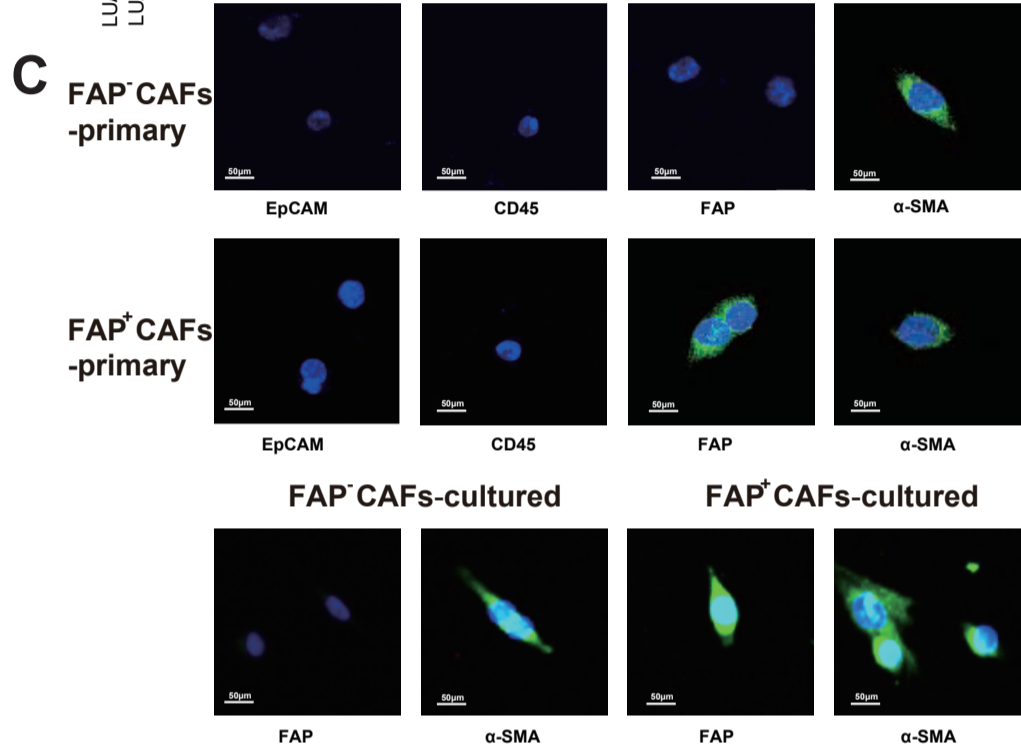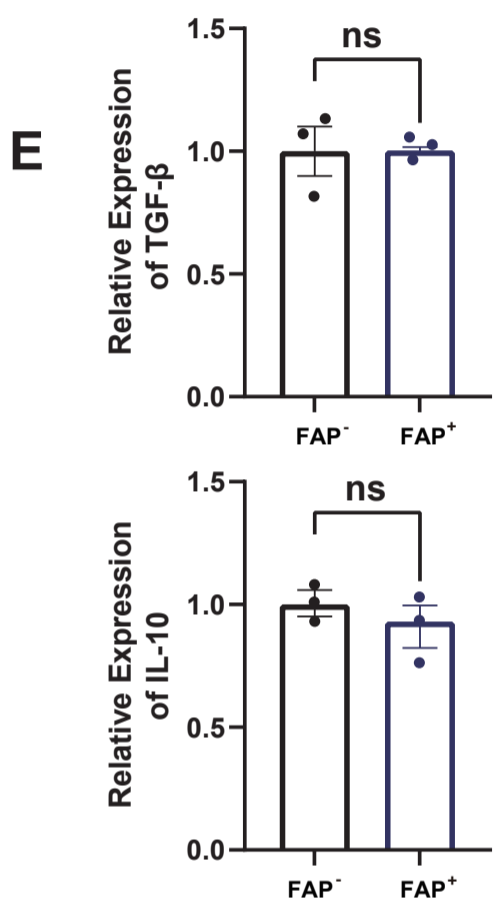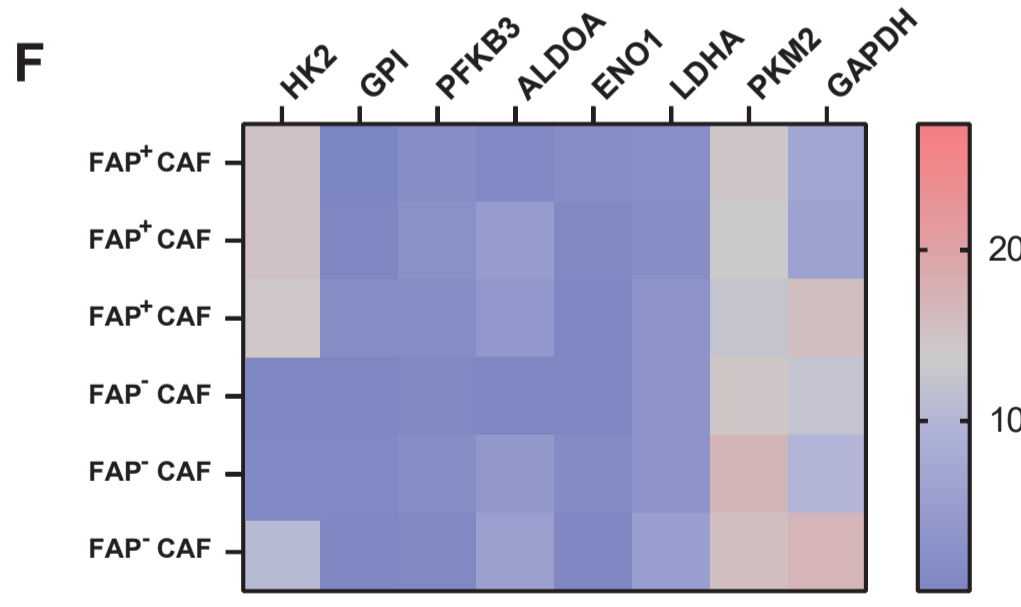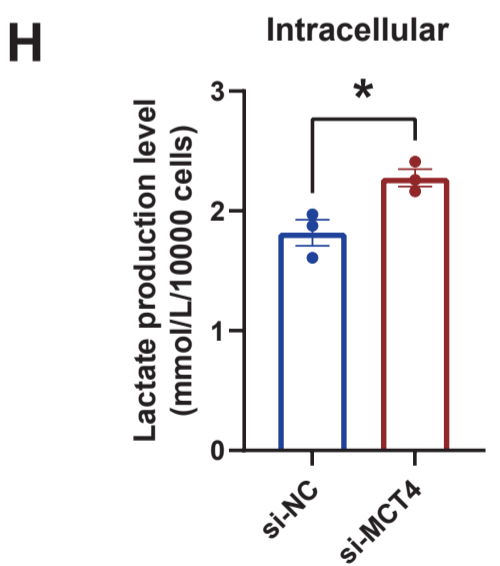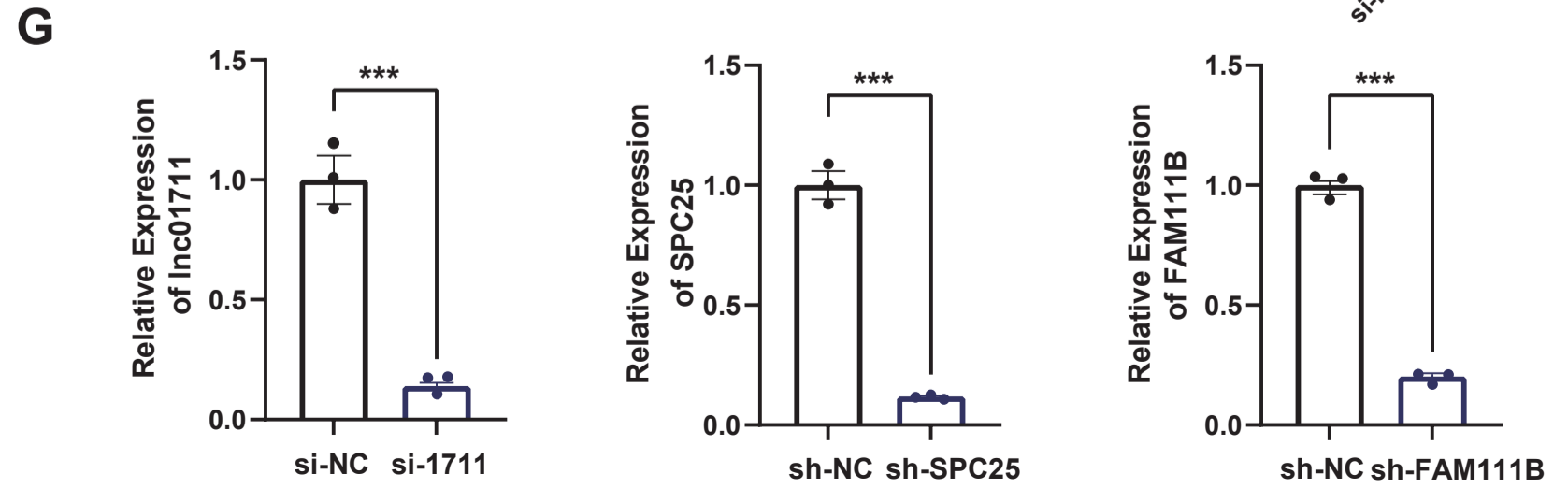

Supplement: Supplementary file 1 — Figure S1 [file 41419_2025_7974_MOESM1_ESM.pdf]

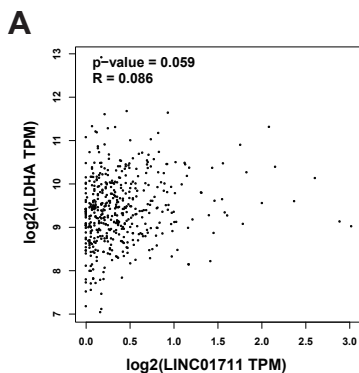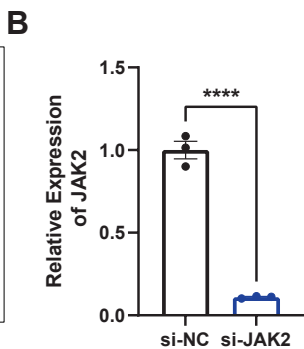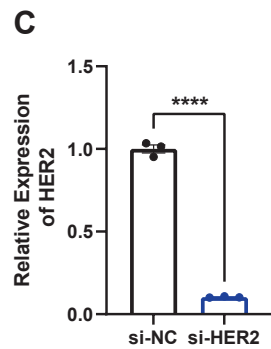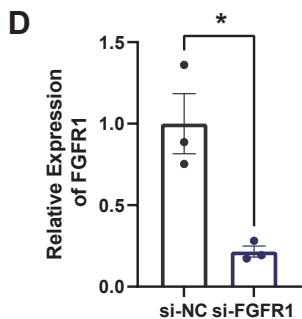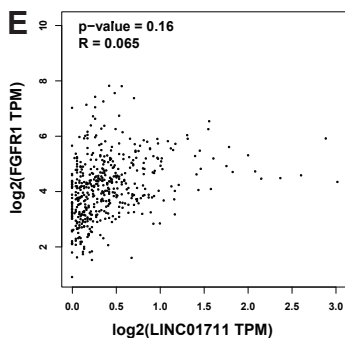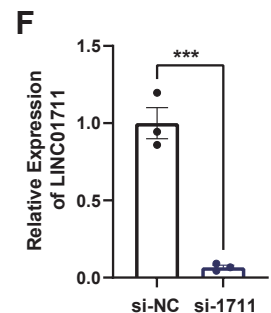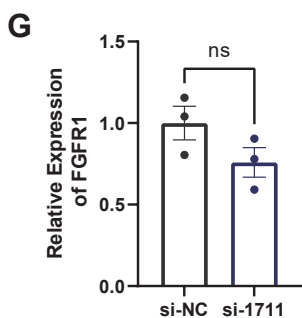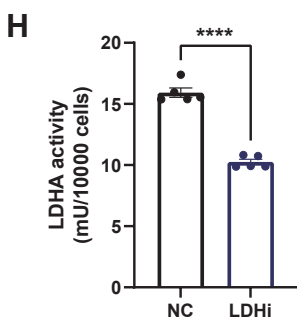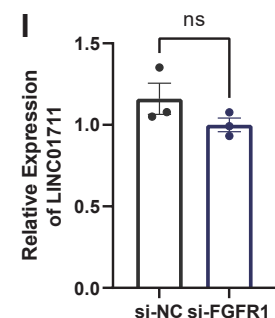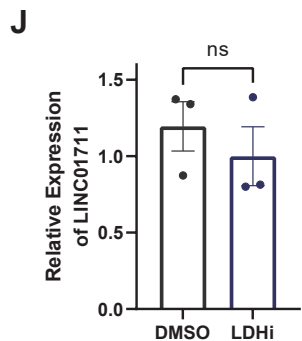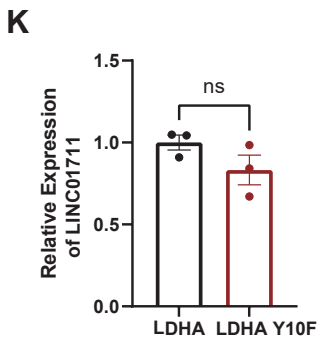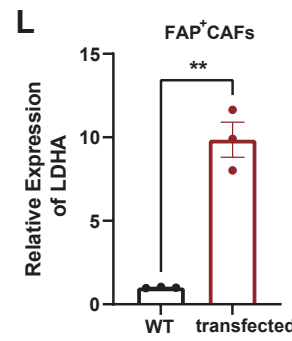

Supplement: Supplementary file 3 — Figure S3 [file 41419_2025_7974_MOESM3_ESM.pdf]

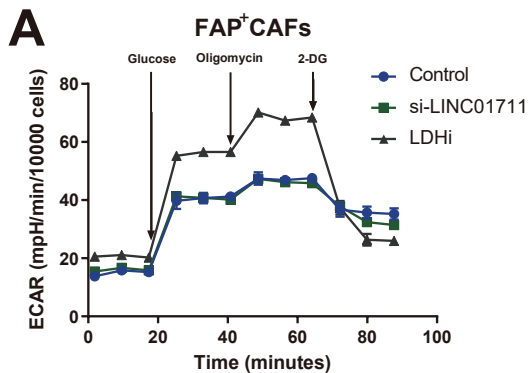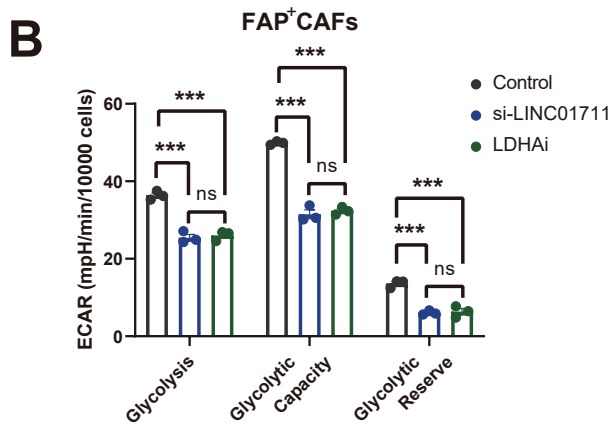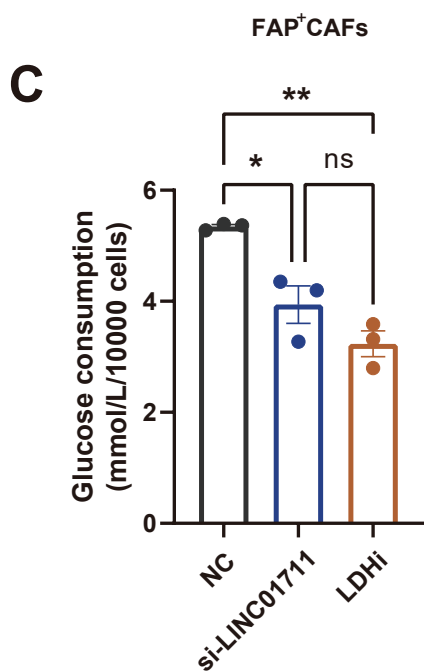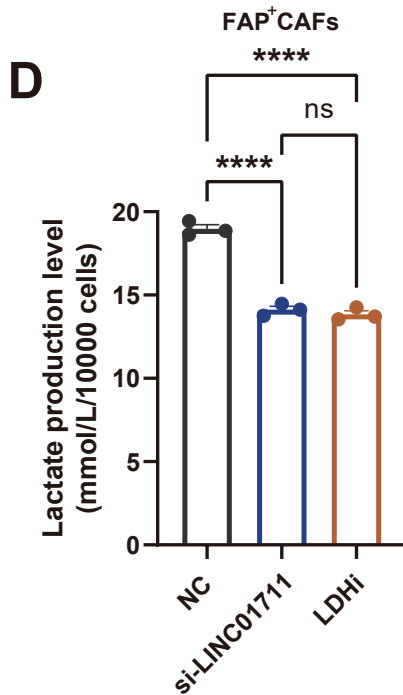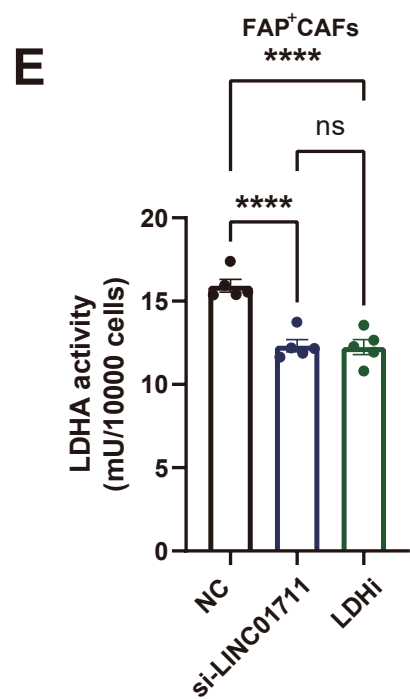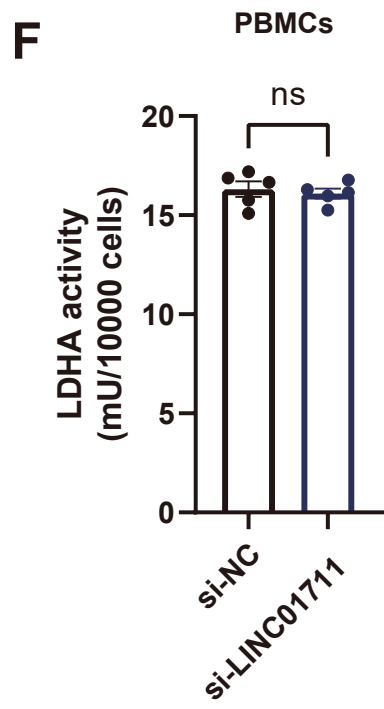

Supplement: Supplementary file 4 — Figure S4 [file 41419_2025_7974_MOESM4_ESM.pdf]

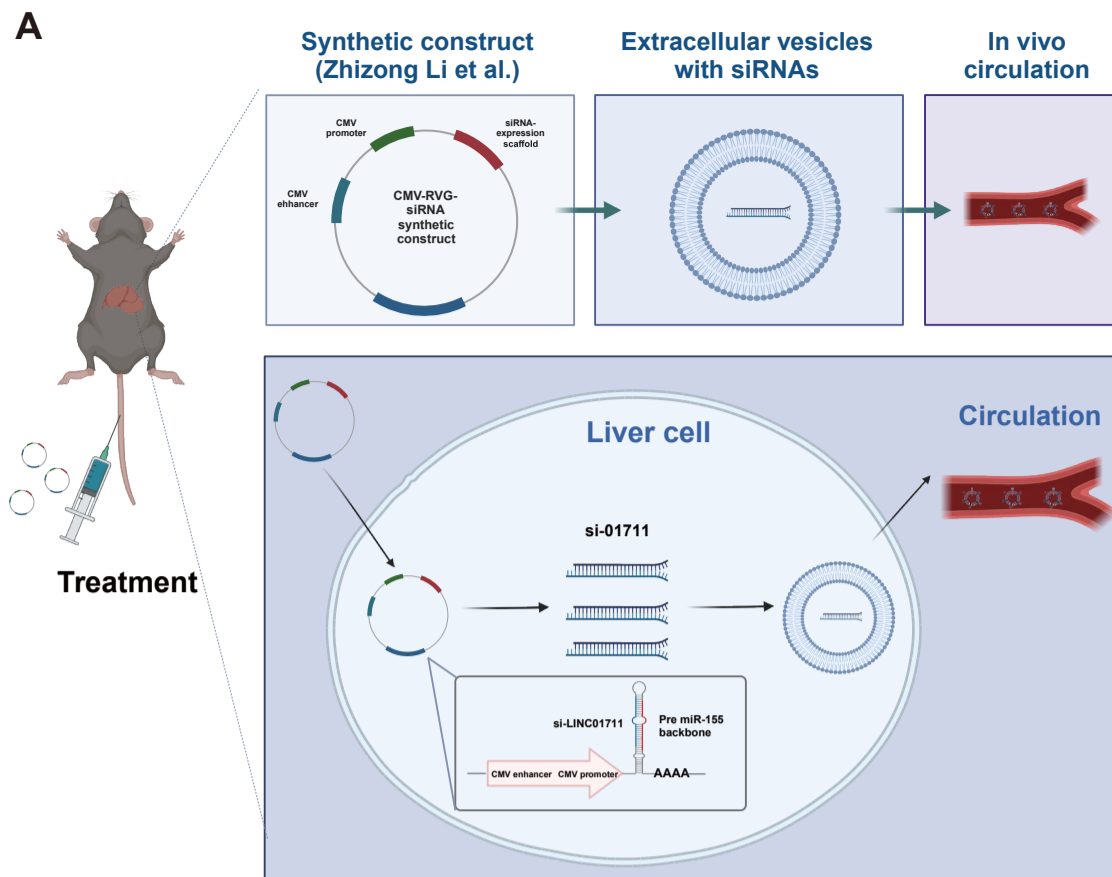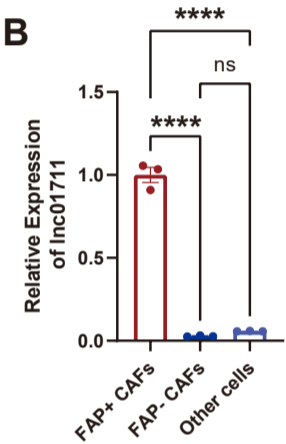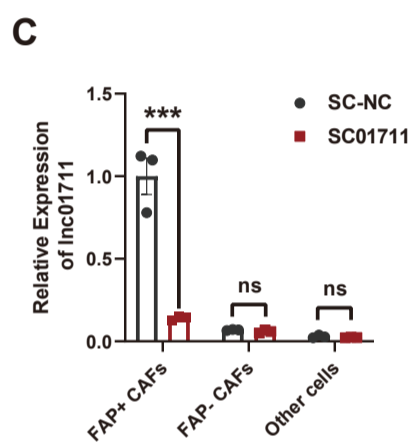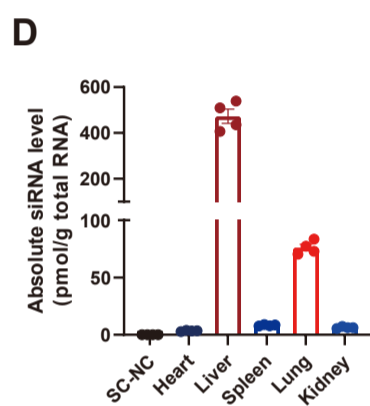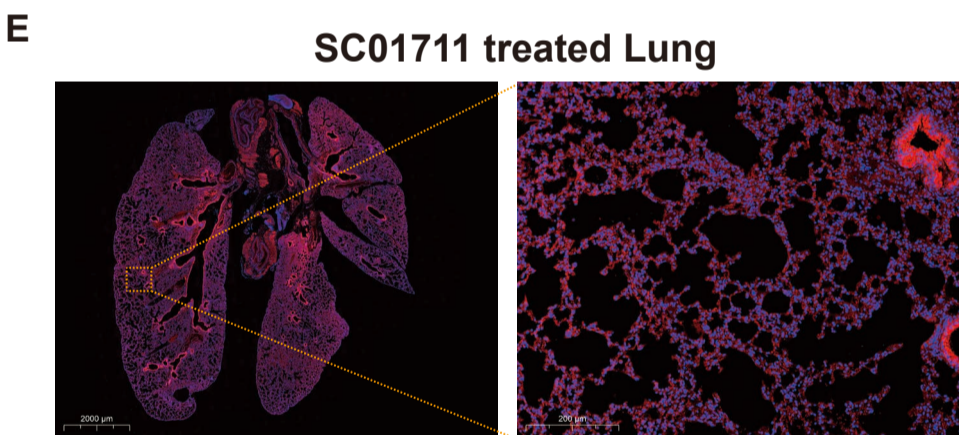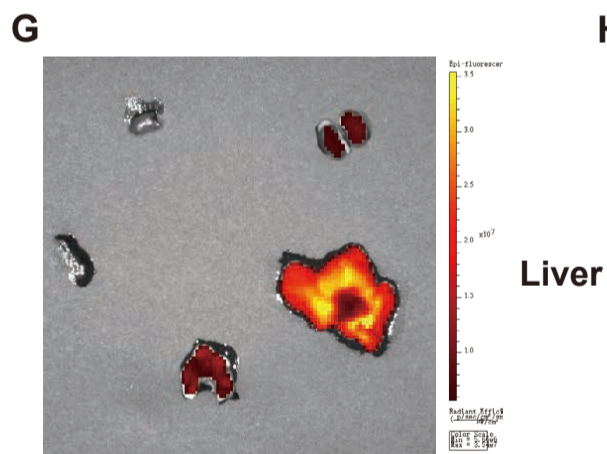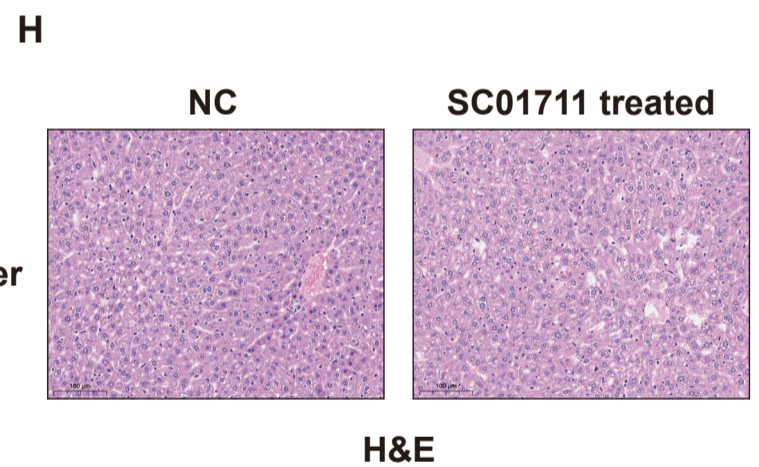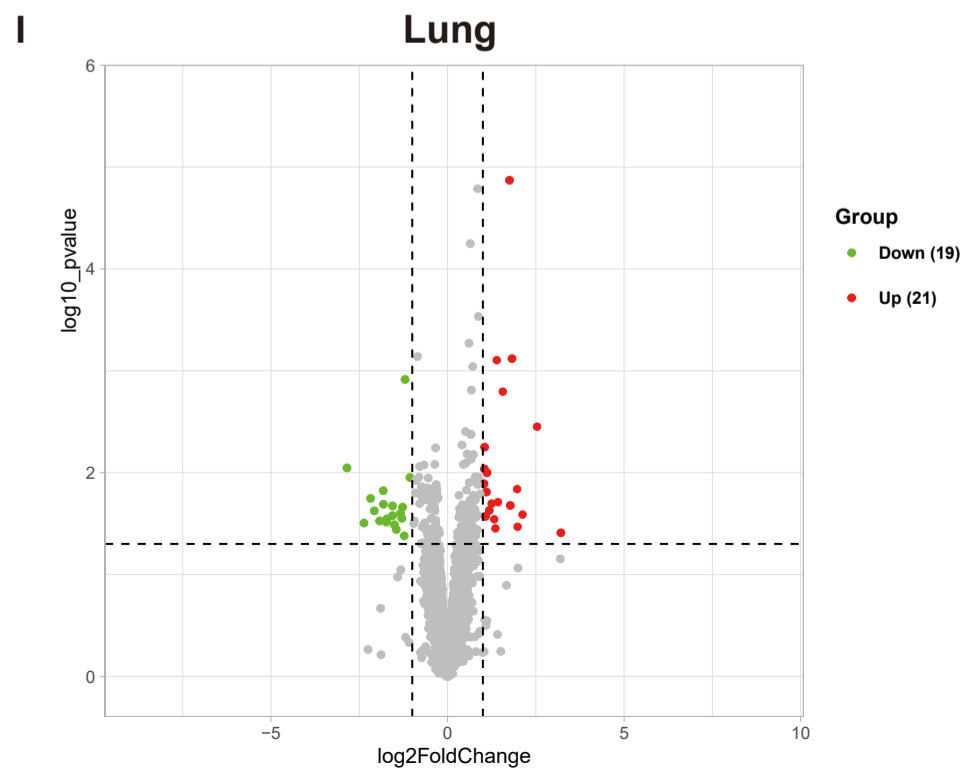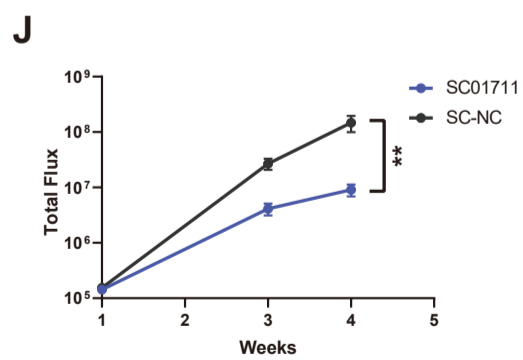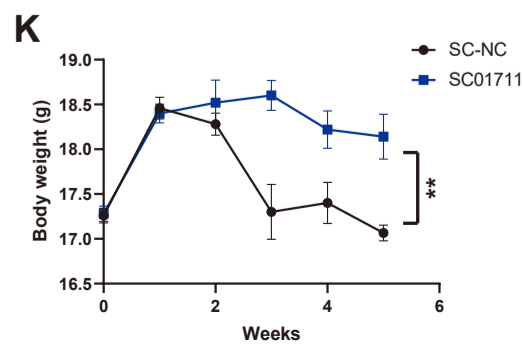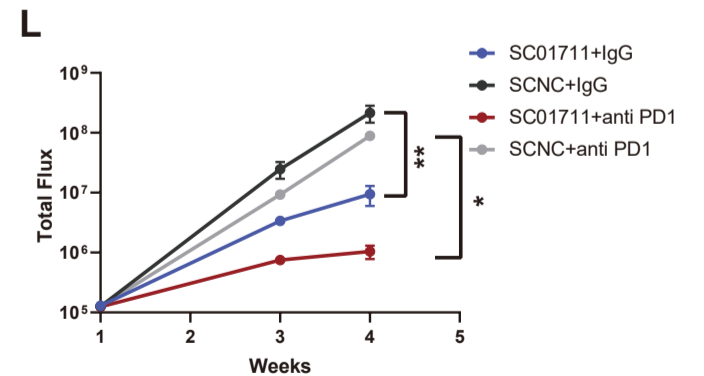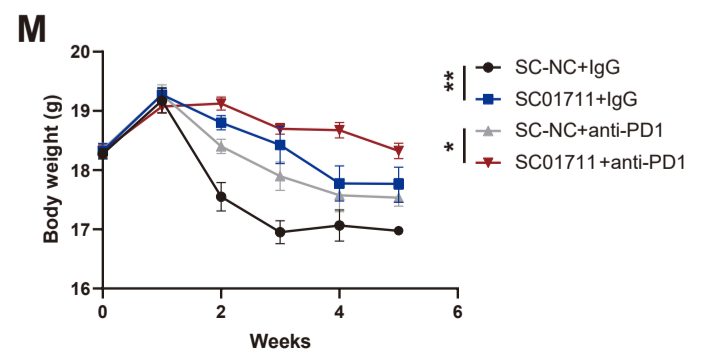

Supplement: Supplementary file 5 — Figure S5 [file 41419_2025_7974_MOESM5_ESM.pdf]
