# Supplementary material for: Targeting LINC01711 in FAP+ cancer-associated fibroblasts overcomes lactate-mediated immunosuppression and enhances anti-PD-1 efficacy in lung adenocarcinoma
Source: Cell Death Dis. 2025 Aug 25;16(1):642. doi: 10.1038/s41419-025-07974-6 (PMC12379239; doi:10.1038/s41419-025-07974-6)
Supplement: Supplementary file 2 — Figure S2 [file 41419_2025_7974_MOESM2_ESM.pdf]

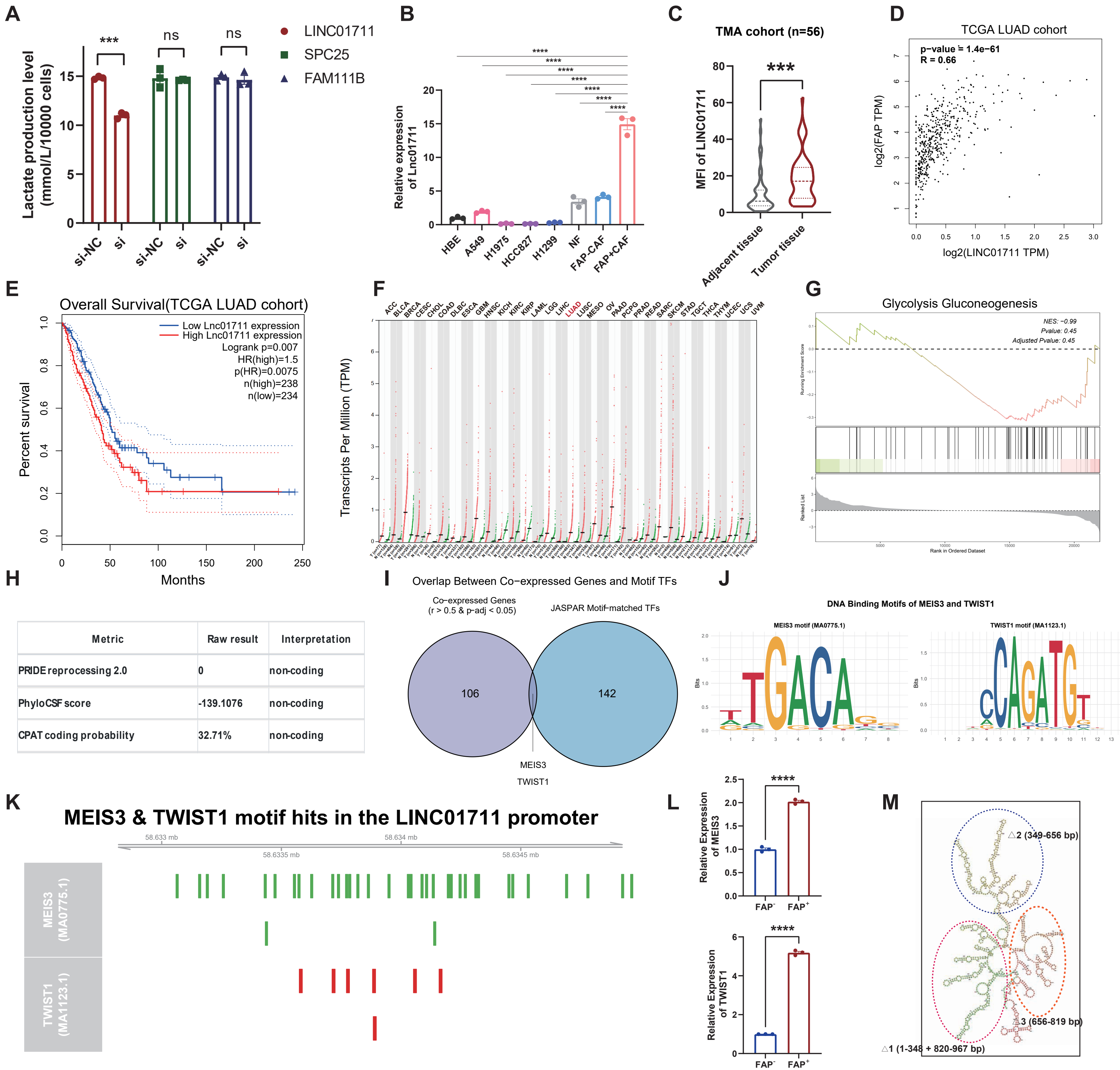
A
Lactate production level (mmol/L/10000 cells)
***
ns
ns
LINC01711
SPC25
FAM111B
si-NC
si
B
Relative expression of Lnc01711
****
HBE
A549
H1975
HCC827
H1299
NF
FAP-CAF
FAP+CAF
C
TMA cohort (n=56)
***
MFI of LINC01711
Adjacent tissue
Tumor tissue
D
TCGA LUAD cohort
p-value = 1.4e−61
R = 0.66
log2(FAP TPM)
log2(LINC01711 TPM)
E
Overall Survival(TCGA LUAD cohort)
Low Lnc01711 expression
High Lnc01711 expression
Logrank p=0.007
HR(high)=1.5
p(HR)=0.0075
n(high)=238
n(low)=234
Percent survival
Months
F
Transcripts Per Million (TPM)
ACC
BLCA
BRCA
CESC
CHOL
COAD
DLBC
ESCA
GBM
HNSC
KICH
KIRC
KIRP
LAML
LGG
LIHC
LUAD
LUSC
MESO
OV
PAAD
PCPG
PRAD
READ
SARC
SKCM
STAD
TGCT
THCA
THYM
UCEC
UCS
UVM
G
Glycolysis Gluconeogenesis
NES: −0.99
Pvalue: 0.45
Adjusted Pvalue: 0.45
Running Enrichment Score
Ranked List
Rank in Ordered Dataset
H
Metric
Raw result
Interpretation
PRIDE reprocessing 2.0
0
non-coding
PhyloCSF score
-139.1076
non-coding
CPAT coding probability
32.71%
non-coding
I
Overlap Between Co−expressed Genes and Motif TFs
Co−expressed Genes
(r > 0.5 & p-adj < 0.05)
JASPAR Motif-matched TFs
106
142
MEIS3
TWIST1
J
DNA Binding Motifs of MEIS3 and TWIST1
MEIS3 motif (MA0775.1)
TWIST1 motif (MA1123.1)
Bits
K
MEIS3 & TWIST1 motif hits in the LINC01711 promoter
58.633 mb
58.634 mb
58.6335 mb
58.6345 mb
MEIS3 (MA0775.1)
TWIST1 (MA1123.1)
L
****
Relative Expression of MEIS3
Relative Expression of TWIST1
FAP⁻
FAP⁺
M
△2 (349-656 bp)
△3 (656-819 bp)
△1 (1-348 + 820-967 bp)
