# Supplementary material for: Targeting LINC01711 in FAP+ cancer-associated fibroblasts overcomes lactate-mediated immunosuppression and enhances anti-PD-1 efficacy in lung adenocarcinoma
Source: Cell Death Dis. 2025 Aug 25;16(1):642. doi: 10.1038/s41419-025-07974-6 (PMC12379239; doi:10.1038/s41419-025-07974-6)
Supplement: Supplementary file 6 — Table S1-S4 and supplement figure legends [file 41419_2025_7974_MOESM6_ESM.docx]

**Table S1. Antibodies, inhibitors and recombinant proteins**

| Antibodies | Source | Catalog number |
| --- | --- | --- |
| BV711 anti-mouse CD3 | Biolegend |  |
| BV510 anti-mouse CD8α | Biolegend |  |
| Percp/cy5.5 anti-mouse CD45 | Biolegend |  |
| PE anti-mouse CD69 | Biolegend |  |
| FITC anti-human GZMB | Biolegend |  |
| BV711 anti-human CD3 | Biolegend |  |
| Percp/cy5.5 anti-human CD45 | Biolegend |  |
| BV510 anti-human CD8 | Biolegend |  |
| FITC anti-human GZMB | Biolegend |  |
| PE anti-human CD69 | Biolegend |  |
| HSP90 Mouse mAb | Proteintech |  |
| MCT4 Rabbit pAb | Proteintech |  |
| β-actin Rabbit pAb | Proteintech |  |
| FGFR1 Rabbit pAb | Proteintech |  |
| Phospho-LDHA(Tyr10) Antibody | Cell signaling technology |  |
| CD8 antibody | Proteintech |  |
| FAP Recombinant antibody | Proteintech |  |
| DYKDDDDK Tag Antibodies  6*His, His-Tag Monoclonal antibody  anti- mouse PD- 1 antibody | Proteintech  Proteintech  BioXcell |  |
| inhibitors |  |  |
| 3-Hydroxybutyric acid  GSK2837808A (LDHi) | MedChemExpress  MedChemExpress |  |
| Recombinant proteins |  |  |
| Recombinant LDHA  Recombinant FGFR1 | Solarbio  Solarbio |  |

| Table S2. Sequences for probes, primers and siRNAs. | | |
| --- | --- | --- |
| Probes | Sequence |  |
| Cy3 probe for LINC01711 | 5’-cy3-CUGUGUGGACUUGGUGGCCCAUAAGAGGCUCUCGGUG 3’ |  |
|  |  |  |
| Primers | F primer | R primer |
| LINC01711 | TGGCTGGAAACACTCACACTACTG | GCCTGCTGCTCTGTGTCCTG |
| U6 | AAAGCAAATCATCGGACGACC | GTACAACACATTGTTTCCTCGGA |
| β-ACTIN | CTCCATCCTGGCCTCGCTGT | GCTGTCACCTTCACCGTTCC |
| FGFR1 | GAACCGCATCAAGGCTGAACT | CACTAAACGACCGTCTTTGGTAT |
| LDHA | CAGTACATCAAGTGTATCATATG | ATTGCAGCTCCTTTTGGATCC |
| JAK2 | TCTGGGGAGTATGTTGCAGAA | AGACATGGTTGGGTGGATACC |
| HER2 | GGAAGTACACGATGCGGAGACT | ACCTTCCTCAGCTCCGTCTCTT |
| SPC25 | AGTACGGACACCTCCTGTCAG | TCTCAACCATTCGTTCTTCTTCC |
| FAM111B | CCAGACAATTCCCAGGATTAGA | TAGCATACCGCCTACCCAGA |
| MESI3 | ATGGCCCGGAGGTATGATGA | GAAGAGCGGGTGTCCATAGA |
| TWIST1 | GTCCGCAGTCTTACGAGGAG | GCTTGAGGGTCTGAATCTTGCT |
|  |  |  |
| Cloning primer | F primer | R primer |
| LINC01711-△1 | TGCTGTGGGGCAGAGTCTCT | TGCTGTGGGGCCGTCCTG |
| LINC01711-△2 | TACAGGAAGTCAAGGTCAGGC | TCTTTCCCACTTGTTTTTATTTCCAA |
| LINC01711-△3 | GTCAGCAGCACCGAGCCG | GTCAGTTTGTTTGGCCCTCGA |
| LINC01711 | TACAGGAAGTCAAGGTCAGGC | TCTTTCCCACTTGTTTTTATTTCCAA |
|  |  |  |
| siRNAs | 5’-3’ sequence |  |
| si-LINC01711 | GUUUGUCACAGUUUAAGGU(dT)(dT)  ACCUUAAACUGUGACAAAC(dT)(dT) |  |
| si-FGFR1 | CCAAGACAGUGAAGUUCAA(dT)(dT)  UUGAACUUCACUGUCUUGG(dT)(dT) |  |
| si-MCT4 | CCUACUCCGUCUACCUCUU(dT)(dT) AAGAGGUAGACGGAGUAGG(dT)(dT) |  |
| sh-JAK2 | GGAAUGGCCUGCCUUACAA(dT)(dT)  UUGUAAGGCAGGCCAUUCC(dT)(dT) |  |
| si-HER2 | AUUGUCUUCAAUGAGCCGAGC  GCUCGGCUCAUUGAAGACAAU |  |
| sh-FPC25 | GAATTTCAAGAGAATGTAA |  |
| sh-FAM111B | AGCAAAGAAGATGGACACATA |  |
|  |  |  |

| Table S3. The sequence of plasmid designed for BiFC. | |
| --- | --- |
|  | Sequence |
| FGFR-Mvenus_C | TACAGGAAGTCAAGGTCAGGCCATACCCACCACAGTTCGGGCAGCCATAGAGGAGAGCTTTCTTAACGGTCCGCTGGGACCCAAGTGGGCCTCCCCTGGGACTGCGTGCTCCCCTACAGAACCTGATGGCTGGAAACACTCACACTACTGCCTGTCCCGGCATCTCTCCCTCCGCCGCAGGGGCTGATGCTGCCACAGGACACAGAGCAGCAGGCCCCAGAGGCTTCTCTGCACCTCTGCTGCCTTTCTGAGTAATCAGAGGAGGCCACACCCACTCCAGGACACCCACACGGGCCTTCTGGGCGATGACGCTCCTGGCCCAGGAGTGGGGGGCTGCCTCACACCTGCTGCTGTGGGGCAGAGTCTCTCCTGGGTCGGGGCCTCGCTGGCGGATCTGCTCCATGTCGTGTGATGTGCTGCAGCCCCGCGTCACCGAGAGCCTCTTATGGGCCACCAAGTCCACACAGCCACTGGCCATGGGGCACCCTGCAGGGCCTCTGGACACGGAGACCTGTGGTTCTCAGCCAGTGGTGATTTTGTGTCCAGGGAACACCTGGCCAAGTCTGGGTTCAATTTTGTTTGTCACAGTTTAAGGTTGGGGTGTCACTAGACATCCTACAGTGCACAGGACGGCCCCACAGCAGACCAGCCCGACTGTCAGCAGCACCGAGCCGGGAAAGCCTGGTCTGGAGCCGTTTCTCTCTAGGGTCCCTGCTAGGGAACCCCTCACTGCCATGGAGAACCCCTCTCCTCCTGTCTCTCAGGACTCTCCGAGGGTCAAGGATGGATGCCTCCCCATCGAGGGCCAAACAAACTGACAAATCGAGTGGGCAAACGGCTTACTCAAAGGCCAGGAGCAGCTACCCGATGACTGCTCCCTCGACCTCTCTCTTCTCACTTTCTTCCCTATTCCATCCTTTGATGGGTGGCCTGTTACACGTTTGGAAATAAAAACAAGTGGGAAAGA |
| LDHA-Mvenus_N | GCCACCATGGCAACTCTAAAGGATCAGCTGATTTATAATCTTCTAAAGGAAGAACAGACCCCCCAGAATAAGATTACAGTTGTTGGGGTTGGTGCTGTTGGCATGGCCTGTGCCATCAGTATCTTAATGAAGGACTTGGCAGATGAACTTGCTCTTGTTGATGTCATCGAAGACAAATTGAAGGGAGAGATGATGGATCTCCAACATGGCAGCCTTTTCCTTAGAACACCAAAGATTGTCTCTGGCAAAGACTATAATGTAACTGCAAACTCCAAGCTGGTCATTATCACGGCTGGGGCACGTCAGCAAGAGGGAGAAAGCCGTCTTAATTTGGTCCAGCGTAACGTGAACATCTTTAAATTCATCATTCCTAATGTTGTAAAATACAGCCCGAACTGCAAGTTGCTTATTGTTTCAAATCCAGTGGATATCTTGACCTACGTGGCTTGGAAGATAAGTGGTTTTCCCAAAAACCGTGTTATTGGAAGCGGTTGCAATCTGGATTCAGCCCGATTCCGTTACCTAATGGGGGAAAGGCTGGGAGTTCACCCATTAAGCTGTCATGGGTGGGTCCTTGGGGAACATGGAGATTCCAGTGTGCCTGTATGGAGTGGAATGAATGTTGCTGGTGTCTCTCTGAAGACTCTGCACCCAGATTTAGGGACTGATAAAGATAAGGAACAGTGGAAAGAGGTTCACAAGCAGGTGGTTGAGAGTGCTTATGAGGTGATCAAACTCAAAGGCTACACATCCTGGGCTATTGGACTCTCTGTAGCAGATTTGGCAGAGAGTATAATGAAGAATCTTAGGCGGGTGCACCCAGTTTCCACCATGATTAAGGGTCTTTACGGAATAAAGGATGATGTCTTCCTTAGTGTTCCTTGCATTTTGGGACAGAATGGAATCTCAGACCTTGTGAAGGTGACTCTGACTTCTGAGGAAGAGGCCCGTTTGAAGAAGAGTGCAGATACACTTTGGGGGATCCAAAAGGAGCTGCAATTTGGTGGCGGTGGCTCGGGCGGTGGTGGATCTGGTGGCGGCGGATCTATGGTGAGCAAGGGCGAGGAGCTGTTCACCGGGGTGGTGCCCATCCTGGTCGAGCTGGACGGCGACGTAAACGGCCACAAGTTCAGCGTGTCCGGCGAGGGCGAGGGCGATGCCACCTACGGCAAGCTGACCCTGAAGCTGATCTGCACCACCGGCAAGCTGCCCGTGCCCTGGCCCACCCTCGTGACCACCCTGGGCTACGGCCTGCAGTGCTTCGCCCGCTACCCCGACCACATGAAGCAGCACGACTTCTTCAAGTCCGCCATGCCCGAAGGCTACGTCCAGGAGCGCACCATCTTCTTCAAGGACGACGGCAACTACAAGACCCGCGCCGAGGTGAAGTTCGAGGGCGACACCCTGGTGAACCGCATCGAGCTGAAGGGCATCGACTTCAAGGAGGACGGCAACATCCTGGGGCACAAGCTGGAGTACAACTACAACAGCCACAACGTCTATATCATGGCCTAA |

| Table S4. The interacted protein candidates of LINC01711 detected by LS/MS. | | | |
| --- | --- | --- | --- |
| Gene names | Number of proteins | Peptides | Score |
| SEPT7 | 15 | 1 | 6.9075 |
| A5CLH5_HUMAN Truncated MHC class I antigen | 1 | 1 | / |
| RBM3 | 2 | 1 | 6.8917 |
| SDHA | 7 | 1 | 6.2391 |
| RRAS2;RRAS | 5 | 1 | 5.7363 |
| VCL;HEL114 | 9 | 2 | 27.385 |
| KDELR2;KDELR1 | 5 | 1 | 5.6581 |
| TXNDC5;STRF8;DKFZp666I134 | 5 | 2 | 12.7 |
| H2AFJ | 20 | 1 | 5.832 |
| RHOC;RHOA | 20 | 1 | 5.8724 |
| VASP | 10 | 3 | 18.526 |
| YWHAH | 5 | 2 | 6.8949 |
| APOL2 | 4 | 1 | 6.2391 |
| MYH9 | 28 | 16 | 114.37 |
| XRCC6 | 5 | 1 | 5.966 |
| LASP1 | 7 | 2 | 12.074 |
| ACLY;ACLY variant protein | 5 | 1 | 5.9803 |
| RAB5C | 3 | 1 | 10.23 |
| FKBP10;DKFZp666D193 | 5 | 1 | 6.3534 |
| VAT1 | 4 | 1 | 5.8599 |
| HNRPK;HNRNPK | 9 | 4 | 24.311 |
| RPL17;hCG_24487;RPL17-C18orf32 | 11 | 1 | 6.8027 |
| TMEM43 | 3 | 1 | 6.2013 |
| CTNNB1 | 14 | 1 | 6.7158 |
| RPL15 | 7 | 1 | 6.0729 |
| EXOSC7 | 4 | 1 | 5.7846 |
| MAP4 | 11 | 5 | 31.59 |
| BSN | 2 | 1 | 6.6429 |
| FLNB;DKFZp686A1668 | 6 | 4 | 14.057 |
| RPL29 | 4 | 1 | 5.7727 |
| PARP1 | 5 | 1 | 5.9308 |
| HSPD1 | 10 | 1 | 5.6639 |
| HSPE1;EPFP1 | 6 | 1 | 6.8548 |
| IDH1;HEL-216 | 10 | 2 | 15.507 |
| HDLBP | 19 | 1 | 5.9364 |
| ENO1 | 21 | 3 | 18.49 |
| MDH2 | 5 | 1 | 8.9698 |
| ZNF577 | 3 | 1 | 5.874 |
| hCG_2004714;FTH1 | 11 | 1 | 5.935 |
| TNKS1BP1 | 3 | 1 | 7.3873 |
| SF1 | 9 | 1 | 5.8256 |
| SERPINH1 | 13 | 2 | 16.091 |
| PICALM;MLL/CALM fusion | 9 | 1 | 5.8307 |
| CTTN | 6 | 1 | 6.1954 |
| HEL-S-270;ANXA2;ANXA2P2 | 28 | 10 | 77.428 |
| RAB11A;RAB11B | 6 | 1 | 6.5792 |
| PPP4C | 8 | 1 | 6.1418 |
| ACTN1;ACTN4 | 23 | 1 | 9.8586 |
| NPC2 | 10 | 1 | 7.0158 |
| DLST;E2k | 12 | 2 | 12.187 |
| ENAH;EVL | 9 | 1 | 6.3121 |
| MT1X;MT1M;MT1E;MT2A;MT1G | 9 | 1 | 7.8275 |
| DLD | 5 | 2 | 12.899 |
| CALU | 5 | 1 | 6.1386 |
| CAV1 | 10 | 3 | 18.504 |
| AKR1B1 | 6 | 1 | 5.8912 |
| LOC115098;CCDC124 | 2 | 1 | 6.8061 |
| CLTB | 3 | 1 | 6.2668 |
| RPL7 | 2 | 1 | 5.7143 |
| EIF3B;EIF3S9 | 6 | 1 | 10.693 |
| ENG | 4 | 1 | 5.8055 |
| STOM | 4 | 2 | 15.113 |
| SPTAN1 | 10 | 3 | 19.455 |
| NPLOC4 | 3 | 1 | 6.0295 |
| SUMO2;SUMO3;SUMO4 | 6 | 2 | 14.824 |
| P4HB | 33 | 3 | 21.353 |
| EIF4A3;DDX48 | 3 | 1 | 6.5186 |
| PABPC1;PABPC3 | 18 | 1 | 6.0934 |
| MTDH | 4 | 1 | 7.4775 |
| RPL30 | 4 | 1 | 6.1518 |
| NDRG1;TRG14 | 13 | 1 | 43.643 |
| HUWE1 | 5 | 1 | 7.5534 |
| MAGED2 | 4 | 1 | 5.8305 |
| HIBADH | 2 | 1 | 5.854 |
| CDC42;RHOQ;RAC2;RHOJ;ARHG;RAC1;HEL-S-42;RHOG;RAC3 | 33 | 2 | 12.971 |
| ACTR3;ACTR3B | 11 | 1 | 5.855 |
| HRAS;K-RAS;KRAS;c-bas/has;NRAS | 24 | 1 | 5.9287 |
| HNRNPA1;HNRPA1;hCG_2020860 | 20 | 2 | 11.61 |
| COPZ1 | 9 | 1 | 6.1409 |
| CS | 12 | 1 | 5.8825 |
| RAP1B;RAP1A | 13 | 1 | 5.6504 |
| SHMT2;DKFZp686P09201;HEL-S-51e | 14 | 1 | 5.7098 |
| RAB21 | 2 | 1 | 19.766 |
| CSRP2 | 4 | 1 | 7.0158 |
| CKAP4 | 3 | 3 | 22.864 |
| TXNRD1 | 15 | 1 | 6.3121 |
| CAMKK2 | 7 | 1 | 5.6685 |
| DSG3 | 2 | 1 | 6.0486 |
| hCG_1641044;VAPA | 3 | 1 | 5.8825 |
| IQGAP1;hCG_1991735 | 9 | 1 | 6.133 |
| RNH1 | 9 | 1 | 7.0158 |
| CD151 | 8 | 1 | 5.8865 |
| NAP1L4 | 12 | 1 | 7.0968 |
| CARS | 8 | 1 | 7.0158 |
| hCG_1821276;PPP1R18 | 7 | 1 | 5.8924 |
| VARS | 11 | 1 | 5.6586 |
| HSP90AB1;HSP90AB2P | 9 | 3 | 42.548 |
| LIM;PDLIM5 | 5 | 1 | 6.03 |
| KPNA3 | 3 | 1 | 5.9662 |
| V5NSX3 | 2 | 2 |  |
| Tcr-alpha;TRAJ56 | 2 | 1 | 5.9972 |
| TAF15 | 4 | 1 | 6.1836 |
| TRA1;HEL-S-125m;HSP90B2P;HSP90B1 | 9 | 1 | 5.6731 |
| FKBP12-Exin;FKBP1A | 7 | 1 | 9.915 |
| GLIPR2;C9orf19 | 4 | 1 | 5.7994 |
| SPTBN1 | 6 | 2 | 14.114 |
| SMTN | 11 | 1 | 6.773 |
| CLTC | 2 | 1 | 7.4801 |
| ILK;HEL-S-28 | 7 | 1 | 5.6634 |
| PSMD1 | 18 | 2 | 11.919 |
| RUNX1;RUNX1T1;CBFA2T3 | 14 | 1 | 5.6461 |
| TPM3;DKFZp686J1372;TPM3-ROS1 | 20 | 9 | 45.913 |
| DKFZp686G2045;ADRM1 | 3 | 1 | 5.774 |
| RAVER1 | 4 | 1 | 9.8586 |
| YTHDF3 | 5 | 1 | 6.3652 |
| DKFZp686A1195;TJP1;DKFZp686M05161 | 11 | 2 | 11.718 |
| SNX12 | 3 | 1 | 5.7231 |
| SLC3A1 | 7 | 1 | 5.8006 |
| HNRNPM | 8 | 1 | 16.213 |
| PSME2 | 8 | 1 | 5.6892 |
| ERP70;PDIA4 | 3 | 3 | 22.67 |
| UQCRH;UQCRHL | 3 | 1 | 15.992 |
| RPL13A | 9 | 1 | 8.1675 |
| EPB41L3 | 10 | 1 | 6.9968 |
| PCMT1 | 6 | 1 | 5.6737 |
| GSN | 8 | 1 | 6.5187 |
| TOR1AIP1 | 4 | 1 | 5.7725 |
| NUDC;NPD011 | 4 | 2 | 11.66 |
| ATP1A1;ATP1A2;ATP1A3;ATP1A4;ATP12A | 27 | 1 | 5.8573 |
| NCEH1 | 3 | 1 | 5.9336 |
| TTN | 5 | 1 | 7.1396 |
| TNFSF13;TNFSF12-TNFSF13 | 8 | 1 | 7.1153 |
| HTRA2 | 4 | 1 | 5.7873 |
| CAST | 45 | 6 | 37.222 |
| ALB | 13 | 2 | 13.026 |
| PTBP1;ROD1;PTBP3 | 11 | 2 | 13.625 |
| FXR1 | 8 | 1 | 6.4405 |
| LRRFIP1 | 3 | 3 | 18.613 |
| TSTA3 | 8 | 1 | 6.2621 |
| HEL107;TKT | 8 | 1 | 5.7154 |
| USH2A | 2 | 1 | -2 |
| HBA2;HBA1 | 23 | 1 | 6.8007 |
| ALDOA;HEL-S-87p | 5 | 1 | 7.776 |
| ANXA6 | 12 | 1 | 6.7348 |
| CLPTM1 | 4 | 1 | 7.9051 |
| CRK | 5 | 1 | 6.3314 |
| ATP2A2 | 3 | 1 | 5.6504 |
| KRT6A;KRT6C | 17 | 6 | 32.551 |
| NPM1 | 14 | 1 | 5.9871 |
| HCC5;MCM3 | 6 | 1 | 8.379 |
| CNBP | 3 | 1 | 5.7404 |
| YARS | 3 | 1 | 5.8748 |
| HEL-S-182mP;GNMT | 3 | 1 | 5.9957 |
| HM13 | 7 | 2 | 11.709 |
| FASN | 2 | 1 | 5.7089 |
| DYNLRB2;DYNLRB1 | 7 | 1 | 6.8604 |
| FGG | 4 | 1 | 8.8852 |
| GLRX3 | 2 | 1 | 28.826 |
| GPD2 | 5 | 1 | 5.892 |
| PXN | 9 | 1 | 6.1654 |
| DARS | 5 | 1 | 5.7702 |
| USP5 | 3 | 2 | 24.871 |
| AIFM1 | 9 | 1 | 7.4788 |
| PSMC3 | 9 | 1 | 6.0514 |
| SND1 | 8 | 5 | 32.263 |
| TALDO1 | 3 | 2 | 13.573 |
| ATP6V1B2 | 4 | 1 | 5.8161 |
| CALD1 | 12 | 3 | 20.184 |
| QDPR | 5 | 1 | 7.7548 |
| UAP1 | 2 | 2 | 11.775 |
| DNCI2;DYNC1I2 | 16 | 1 | 5.7956 |
| KRT10 | 2 | 5 | 69.089 |
| DPYSL2 | 5 | 1 | 6.2668 |
| CYGB | 3 | 1 | 6.0729 |
| TLN1 | 2 | 4 | 28.567 |
| KCNC1 | 4 | 1 | 5.6958 |
| GPI;SIX5 | 6 | 1 | 5.667 |
| UBB;UBC;UBA52;RPS27A;HEL112;DKFZp434K0435;UbC | 39 | 1 | 5.8731 |
| CHCHD3 | 6 | 1 | 6.1391 |
| NDUFS1 | 5 | 1 | 5.6832 |
| HBD;HBG2;HBG1;HBE1 | 62 | 1 | 5.671 |
| HADHA | 6 | 1 | 8.3557 |
| ATXN2 | 17 | 1 | 6.3072 |
| SHROOM3 | 5 | 1 | 5.804 |
| DYNC1H1 | 30 | 2 | 11.328 |
| HEL-S-108;TPM4 | 15 | 8 | 27.237 |
| RPS14 | 3 | 2 | 12.416 |
| RPS24 | 4 | 1 | 6.1418 |
| ARHGEF11 | 2 | 1 | -2 |
| DCTN2;HEL-S-77 | 16 | 2 | 11.695 |
| GRB2 | 5 | 1 | 5.6603 |
| FSCN1 | 3 | 2 | 13.502 |
| P3.58;ICAM1;sICAM-1 | 10 | 2 | 11.609 |
| LMNA | 14 | 2 | 12.041 |
| LGALS1 | 3 | 3 | 17.676 |
| CAPZB | 6 | 1 | 7.2185 |
| TAGLN2 | 5 | 3 | 20.055 |
| RUVBL1 | 3 | 1 | 7.2439 |
| PCBP2 | 7 | 2 | 7.0047 |
| NAGK | 4 | 2 | 15.93 |
| EEF2 | 8 | 4 | 23.569 |
| CSE1L | 6 | 1 | 6.1695 |
| HSPA7;HSPA6;HSPA8;HEL-S-103;HSPA1L;HSPA1B;HSPA1A | 28 | 2 | 7.4823 |
| G6PD | 13 | 3 | 18.733 |
| PDIA6 | 2 | 1 | 8.5754 |
| HDGF | 4 | 1 | 6.8458 |
| LMAN2 | 4 | 1 | 5.7023 |
| MYL9;MYL12A;MYL12B | 9 | 2 | 11.678 |
| AP3M2 | 3 | 1 | 5.6792 |
| TUBB;TUBB2B;TUBB2A;XTP3TPATP1;TUBB2C | 52 | 4 | 29.792 |
| HSPA9;HEL-S-124m | 12 | 2 | 8.3908 |
| CAT | 3 | 1 | 5.9855 |
| KHSRP | 6 | 1 | 6.2918 |
| CD44 | 9 | 3 | 23.912 |
| COPA | 7 | 1 | 6.5017 |
| PGM1 | 5 | 1 | 5.774 |
| TRIM25 | 4 | 1 | 6.773 |
| SEC16A | 7 | 1 | 6.1906 |
| TBCB | 4 | 1 | 7.0158 |
| EEF1A1;EEF1A1P5;EEF1A2;EEF1A;EEF1A1L14 | 77 | 4 | 25.743 |
| ANXA1 | 30 | 5 | 36.88 |
| HEL-213;IGK@;IGKC | 24 | 1 | 9.7509 |
| ZC3HAV1 | 6 | 1 | 5.7727 |
| LDHB | 3 | 2 | 5.8046 |
| AARS | 14 | 1 | 5.872 |
| HEL-S-102;HSPB1 | 8 | 5 | 36.948 |
| GARS | 6 | 1 | 6.1954 |
| ITGB1 | 7 | 4 | 25.878 |
| NCL | 15 | 3 | 17.721 |
| G3BP;DKFZp686L1159;G3BP1 | 12 | 1 | 5.887 |
| KPNA4 | 6 | 1 | 5.8238 |
| EIF3I | 23 | 2 | 14.498 |
| NUDCD1 | 4 | 1 | 5.6584 |
| HNRNPA2B1;HNRPA2B1 | 10 | 2 | 11.923 |
| VCP | 13 | 2 | 13.206 |
| RPL19 | 6 | 1 | 5.8748 |
| CANX | 15 | 2 | 11.829 |
| HEL-S-89n;HSPA5 | 4 | 4 | 76.973 |
| FGF18 | 2 | 1 | 5.9513 |
| EMD | 3 | 1 | 5.8734 |
| ADD1;ADDA | 7 | 1 | 6.5312 |
| HEL-S-269;PDIA3 | 21 | 5 | 39.564 |
| NDUFB11 | 5 | 1 | 6.4508 |
| ERGIC1 | 3 | 1 | 5.8748 |
| PLEC | 10 | 19 | 118.45 |
| MYH10 | 2 | 3 | 5.7404 |
| KRT83;KRT86;KRT81 | 5 | 1 | 20.575 |
| RPL34 | 2 | 2 | 11.637 |
| ARPC1B | 2 | 1 | 6.3844 |
| GAPDH;HEL-S-162eP | 7 | 1 | 87.69 |
| CEP170 | 7 | 1 | 5.8105 |
| PGP | 1 | 1 | 6.7268 |
| USP14 | 5 | 1 | 5.7297 |
| BGN | 9 | 1 | 6.2621 |
| UCHL1;HEL-117 | 5 | 1 | 5.6882 |
| CPNE1 | 19 | 1 | 5.8105 |
| RRBP1 | 7 | 12 | 323.31 |
| HEL-S-273;TPM2b;TPM2;TPM1 | 20 | 10 | 108.4 |
| TUBA1C;TUBA1A;TUBA1B;TUBA2;TUBA4A | 37 | 3 | 23.103 |
| ALK_K17;A20;KIF5C;KIF5A | 40 | 4 | 25.679 |
| GLS | 3 | 1 | 7.8432 |
| PSMC4 | 2 | 1 | 6.7034 |
| HK2;DKFZp686M1669 | 5 | 1 | 7.4779 |
| ENO2 | 4 | 2 | 7.2613 |
| CCT4 | 5 | 3 | 36.171 |
| ACTA2;ACTG2;ACTA1;ACTC1 | 36 | 8 | 6.2668 |
| PHB;HEL-S-54e | 9 | 1 | 6.2591 |
| HEL-S-69p;PPIA | 10 | 2 | 39.165 |
| RPL13 | 4 | 1 | 6.1454 |
| RPS3A | 6 | 2 | 12.429 |
| TIAL1 | 7 | 1 | 6.3649 |
| SUGT1 | 3 | 1 | 9.1018 |
| FARSB | 4 | 1 | 6.2845 |
| SEC24D | 4 | 1 | 5.6832 |
| EIF4A2;EIF4A1 | 7 | 1 | 5.8371 |
| EEA1 | 2 | 1 | 5.671 |
| NUCB1 | 6 | 1 | 13.119 |
| EIF3L | 7 | 1 | 5.855 |
| EIF3D | 4 | 1 | 5.886 |
| PEA15 | 4 | 1 | 7.0157 |
| ARPC5 | 3 | 1 | 6.8804 |
| RPL18A | 11 | 1 | 5.7949 |
| RPS28 | 2 | 2 | 14.444 |
| S100A6 | 3 | 1 | 5.7578 |
| HEL-S-43;S100A11 | 3 | 1 | 5.9855 |
| PLOD1 | 3 | 1 | 5.8376 |
| PRKAR1A;DKFZp779L0468;PRKAR1A/RARA fusion;RET/PTC2 | 17 | 2 | 14.502 |
| SLC9A3R1 | 4 | 1 | 6.7723 |
| HEL-S-105;EZR | 6 | 1 | 5.6465 |
| ATIC;HEL-S-70p | 5 | 1 | 5.7354 |
| PLANH2;SERPINB2 | 6 | 1 | 6.28 |
| TRIM28 | 3 | 1 | 6.2962 |
| HEL-S-21;GSTO1 | 5 | 2 | 11.851 |
| FKBP4 | 3 | 1 | 5.8748 |
| NCLN | 2 | 1 | 6.0729 |
| PSMB7 | 6 | 3 | 18.095 |
| NT5E | 5 | 1 | 12.767 |
| KPNB1 | 3 | 1 | 6.6182 |
| BHMT;HEL-S-61p | 5 | 1 | 6.224 |
| PRMT5 | 8 | 1 | 8.5736 |
| CALM2;CALM3;CALM1;TNNC2;CALML3 | 15 | 2 | 13.086 |
| CAP1 | 9 | 2 | 13.232 |
| EBF2 | 3 | 1 | 6.4638 |
| PALLD;KIAA0992;DKFZp586L0518;MYPN | 8 | 2 | 12.478 |
| TUBB2C;TUBB4B | 6 | 3 | 8.236 |
| YWHAG | 4 | 4 | 19.457 |
| ZMPSTE24 | 2 | 1 | 6.2621 |
| RAP1GDS1 | 5 | 1 | 7.57 |
| PTRF | 4 | 1 | 5.8773 |
| HSPA8;HEL-S-72p;HSPA2 | 27 | 6 | 51.727 |
| SHF | 2 | 1 | 5.8258 |
| SF3B4 | 3 | 1 | 6.2358 |
| VAPB | 6 | 1 | 5.6875 |
| CCT3 | 6 | 1 | 5.7956 |
| SPCS2 | 4 | 1 | 8.5472 |
| MVP | 20 | 1 | 6.2758 |
| SLC1A5 | 5 | 1 | 6.1151 |
| PLS3 | 6 | 1 | 5.7377 |
| HEL-S-5a;HSPA4 | 9 | 1 | 7.4816 |
| PLOD2 | 3 | 2 | 11.687 |
| HEL-S-45;TGM2 | 7 | 5 | 36.36 |
| UBE2K | 4 | 1 | 5.9532 |
| KIAA1407 | 4 | 1 | 6.1895 |
| GANAB | 3 | 2 | 12.027 |
| LDHA | 19 | 4 | 131.29 |
| IGF2BP2 | 3 | 1 | 6.4943 |
| DKFZp564A2282;PSMD2 | 4 | 1 | 6.9965 |
| DKFZp547C166;LEPRE1 | 3 | 1 | 6.8676 |
| EPRS | 2 | 1 | 5.7183 |
| TUBB6 | 10 | 2 | 5.8374 |
| ANXA11 | 6 | 1 | 8.5435 |
| ARHGAP1 | 4 | 1 | 5.8272 |
| ZYX | 9 | 4 | 26.483 |
| EIF3M | 4 | 1 | 6.0315 |
| PSMD11 | 3 | 1 | 12.404 |
| HEL-S-49;TPI1 | 5 | 1 | 6.9968 |
| UQCRC1 | 2 | 1 | 5.7769 |
| GNB2L1 | 17 | 1 | 6.4377 |
| NRBP1 | 3 | 1 | 5.8734 |
| EIF4G2 | 12 | 1 | 6.2391 |
| COL6A3;DKFZp686K04147 | 6 | 2 | 11.716 |
| BTBD7 | 3 | 1 | 6.2259 |
| CFL1;HEL-S-15 | 9 | 4 | 124.35 |
| HEL-S-78p;FGB | 4 | 3 | 66.943 |
| DSP;DSP variant protein | 4 | 1 | 6.7158 |
| AKAP2 | 2 | 1 | 6.7009 |
| PS1TP5BP1;ACTB | 51 | 11 | 323.31 |
| HEL-S-1;YWHAB | 7 | 3 | 7.0968 |
| U2AF2 | 2 | 1 | 6.0879 |
| RAN | 9 | 3 | 22.195 |
| CCT7 | 5 | 1 | 5.9341 |
| SIN3B | 3 | 1 | 5.8174 |
| STMN2 | 3 | 1 | 6.4106 |
| CCT2;HEL-S-100n | 5 | 1 | 6.0575 |
| AHSG | 3 | 1 | 8.4839 |
| KRT85 | 3 | 1 | 6.6403 |
| PTMA;PTMAP7 | 19 | 4 | 25.716 |
| BCAP31 | 7 | 1 | 5.6737 |
| EEF1E1;EEF1E1-BLOC1S5 | 3 | 1 | 8.2237 |
| SSR3 | 2 | 1 | 6.2186 |
| BUD31 | 1 | 1 | 5.7069 |
| APEH | 5 | 1 | 5.8239 |
| YWHAZ | 10 | 6 | 50.441 |
| SH3BGRL3;HEL-S-297 | 4 | 2 | 13.359 |
| DKFZp686P17171;SERBP1 | 5 | 2 | 18.03 |
| LETM1 | 2 | 1 | 7.9377 |
| ANXA5;HEL-S-7 | 4 | 1 | 6.0203 |
| TPM1 | 17 | 8 | 11.776 |
| SLC4A1 | 4 | 1 | 6.7113 |
| TIA1 | 5 | 1 | 8.236 |
| TBCA | 6 | 1 | 5.673 |
| FABP5 | 2 | 1 | 8.8085 |
| FLJ10983;PGM2 | 4 | 1 | 6.1004 |
| TCP1 | 5 | 1 | 6.9211 |
| YWHAQ | 3 | 3 | 6.1204 |
| EPB41L2 | 10 | 1 | 7.9269 |
| TIMM8B | 2 | 1 | 6.5186 |
| MAPK3 | 5 | 1 | 10.943 |
| CCDC15 | 2 | 1 | 5.7363 |
| RPS3 | 16 | 4 | 28.852 |
| GEN1 | 3 | 1 | 5.6457 |
| HLA-A | 1 | 1 | 5.916 |
| GOLT1B | 4 | 1 | 5.9795 |
| TMEM258 | 2 | 1 | 6.0774 |
| PHB2 | 7 | 2 | 36.887 |
| DSTN;HEL32 | 3 | 2 | 15.112 |
| UBE2N;HEL-S-71;UBE2NL | 4 | 1 | 7.5623 |
| MYL6;MYL6B | 12 | 2 | 12.751 |
| NAP1L1 | 1 | 1 | 6.1418 |
| TBC1D15 | 5 | 1 | 6.2441 |
| CLTA | 2 | 1 | 5.951 |
| UNC79 | 2 | 1 | 5.6916 |
| ERO1L | 3 | 1 | 9.4244 |
| APEX1 | 4 | 1 | 5.7343 |
| MYOF | 2 | 1 | 7.48 |
| AK1 | 5 | 1 | 5.671 |
| RPL28 | 5 | 1 | 5.8068 |
| SLC9A3R2 | 5 | 1 | 6.1695 |
| APRT | 5 | 1 | 8.2241 |
| KARS | 3 | 1 | 6.456 |
| KRT1 | 6 | 12 | 211.27 |
| KRT1 | 1 | 12 | 7.7094 |
| SOD1;HEL-S-44 | 5 | 1 | 6.8291 |
| TXN | 2 | 4 | 43.651 |
| PFN1 | 2 | 2 | 11.49 |
| MIF | 3 | 2 | 21.722 |
| EIF4A1 | 1 | 1 | 5.7548 |
| RPL22 | 8 | 1 | 5.7725 |
| FARSA;FARSLA | 4 | 1 | 7.6865 |
| PVRL2 | 4 | 1 | 6.3884 |
| CALR;HEL-S-99n | 12 | 2 | 88.459 |
| PARK7;HEL-S-67p | 4 | 1 | 6.729 |
| SNRPD2 | 2 | 2 | 11.413 |
| HSP90AA1;EL52;HSP90AA2P | 7 | 2 | 5.8777 |
| CSF2RB | 1 | 1 | 6.1478 |
| RCOR1 | 1 | 1 | 8.4594 |
| MYDGF | 3 | 2 | 12.305 |
| RPS11 | 3 | 1 | 6.4132 |
| RCN3 | 2 | 1 | 5.8374 |
| ZNF541 | 2 | 1 | 5.9423 |
| RPS16 | 5 | 1 | 5.9861 |
| HEL-S-112;PDLIM1 | 2 | 1 | 5.8432 |
| MLNR | 1 | 1 | 5.9211 |
| SNX3 | 1 | 2 | 12.425 |
| UGDH;ugd;GDH | 9 | 2 | 12.553 |
| EDF1 | 1 | 1 | 6.0077 |
| HEL-S-115;SH3BGRL | 2 | 1 | 5.854 |
| NPM3 | 1 | 1 | 6.6803 |
| KRT33B;KRT33A;KRT31;KRT34 | 6 | 2 | 13.172 |
| SNAP29 | 1 | 1 | 5.7399 |
| HEL-S-68p;PGK1 | 5 | 2 | 12.194 |
| A2M | 1 | 1 | 6.6149 |
| COL1A1 | 6 | 4 | 24.547 |
| KRT14;KRT16;KRT17 | 6 | 2 | 8.179 |
| FGA | 8 | 3 | 17.846 |
| RPN1 | 8 | 3 | 17.878 |
| EIF2S1 | 2 | 1 | 8.4833 |
| KRT16;keratin | 2 | 2 | 13.748 |
| IGF2R | 4 | 2 | 11.342 |
| FGFR1 | 26 | 9 | 114.49 |
| FLNA;FLJ00119 | 21 | 35 | 323.31 |
| DKFZp686P10119;IRP1;HEL60;ACO1 | 5 | 1 | 5.7377 |
| HEL-S-123m;ATP5A1 | 9 | 4 | 32.734 |
| HEL70;MSN | 3 | 5 | 30.539 |
| PSMB6 | 2 | 1 | 7.4827 |
| MAPK1 | 4 | 2 | 15.359 |
| MARCKS | 3 | 3 | 44.812 |
| ATP5D | 1 | 1 | 68.284 |
| DNAJA1 | 1 | 1 | 5.6941 |
| SFN | 1 | 3 | 5.8943 |
| KRT9 | 2 | 7 | 64.394 |
| KRT2 | 1 | 6 | 46.033 |
| ATP6V1E1 | 2 | 1 | 6.9349 |
| MARCKSL1 | 1 | 2 | 13.751 |
| ALDH9A1 | 1 | 1 | 6.2962 |
| NHP2L1 | 2 | 1 | 5.9556 |
| ATP5I | 1 | 1 | 5.8241 |
| SEC61B | 3 | 1 | 6.0934 |
| HEL2;YWHAE;YWHAE/FAM22B fusion;YWHAE/FAM22A fusion | 10 | 3 | 14.994 |
| RPS18 | 1 | 1 | 7.0744 |
| SNRPF | 1 | 1 | 5.8804 |
| RPL7A | 2 | 1 | 5.8127 |
| RPL23A;WUGSC:H_DJ0855D21.2;RPL23AL | 8 | 2 | 11.465 |
| RBX1 | 1 | 1 | 7.9074 |
| RPS21 | 6 | 2 | 21.495 |
| ACTG1 | 12 | 11 | 15.523 |
| BASP1 | 2 | 5 | 39.187 |
| FKBP3 | 2 | 1 | 6.4638 |
| TAGLN | 8 | 3 | 17.412 |
| AKAP12;DKFZp686M0430 | 2 | 2 | 12.063 |
| LRP1 | 2 | 1 | 5.8371 |
| DHX9 | 3 | 1 | 6.6671 |
| AHNAK | 11 | 17 | 104.59 |
| HNRNPA0 | 1 | 1 | 5.7725 |
| UBE2V1 | 1 | 1 | 6.0518 |
| EIF4EBP1 | 1 | 1 | 6.2993 |
| TPBG | 1 | 1 | 5.8993 |
| FLNC | 4 | 14 | 61.058 |
| EIF4H;WBSCR1 | 10 | 3 | 18.632 |
| PPA1;HEL-S-66p | 3 | 1 | 6.3028 |
| PCBP1;PCBP2;PCBP3 | 11 | 2 | 13.364 |
| VAMP3;VAMP2;VAMP1 | 13 | 2 | 12.273 |
| AMBN | 3 | 1 | 6.0729 |
| CALML5 | 2 | 2 | 12.491 |
| CFL2 | 4 | 2 | 6.6797 |
| LEPREL2 | 2 | 1 | 6.1695 |
| HEL-S-132P;PHP14;PHPT1 | 3 | 2 | 14.203 |
| GNN | 1 | 1 | 5.6745 |
| IKBIP | 1 | 1 | 6.2237 |
| GET4 | 1 | 1 | 10.28 |
| CCDC50 | 1 | 1 | 5.8055 |
| MAGEB6 | 1 | 1 | 5.6493 |
| TTLL11 | 1 | 1 | 6.5469 |
| LRRC59 | 1 | 1 | 6.1788 |
| S100A16 | 1 | 1 | 5.9583 |
| RPAP3 | 1 | 1 | 5.7005 |
| INTS10 | 1 | 1 | 5.6472 |
| CMTM6 | 1 | 1 | 5.85 |
| GNG12 | 1 | 1 | 7.4855 |
| ZNF629 | 1 | 1 | 5.7744 |
| TRPV2 | 1 | 1 | 6.1802 |
| COPG1;COPG | 6 | 3 | 25.8 |
| DUSP13 | 1 | 1 | 5.7862 |

**Supplemental Figures legends**

**Figure S1.**

A. The expression of FAP in TCGA-LUAD cohort. Tumor (n=515) vs. Normal (n=59), the p value was determined by two-tailed unpaired Student’s *t* test.

B. The correlation analysis between FAP expression and CAFs infiltration. The p value was determined by Spearman Correlation Analysis.

C. Immunofluorescence validated FAP expression in FAP^+^ CAFs and FAP^-^ CAFs (primary or cultured).

D. Representative images of medium of FAP^+^ CAFs and FAP^-^ CAFs after cultured for 3 days (n=3 biological repeats).

E. Elisa analysis revealed the expression of TGF-β and IL-10 in FAP^+^ CAFs and FAP^-^ CAFs (n=3 biological repeats, the p value was determined by two-tailed unpaired Student’s *t* test).

F. The expression of glycolysis-related gene expression in RNA-sequence data (FAP^+^ CAFs vs FAP^-^ CAFs, n=3 biological repeats).

G. The efficiency validation of si-LINC01711, sh-SPC25 and sh-FAM111B using RT-qPCR (n=3 biological repeats, the *P* value was determined by two-tailed unpaired Student’s *t* test).

H. The intracellular lactate production level significantly decreased in FAP^+^ CAFs transfected si-MCT4 (n=3 biological repeats). The *P* value was calculated by two-tailed unpaired *t* test.

All the results were shown as mean ± S.E.M. * *P*≤0.05, ** *P*≤0.01, *** *P*≤0.001, **** *P*≤0.0001.

**Figure S2.**

A. The lactate production level of FAP^+^ CAFs when knocking down top3 genes in RNA-sequence data using siRNA transfection (n=3 biological repeats). The *P* value was calculated by two-tailed unpaired *t* test.

B. The expression of LINC01711 in LUAD cell lines and CAFs (n=3 biological repeats). The *P* value was calculated by two-tailed unpaired *t* test.

C. The MFI of LINC01711 in tumor tissue and adjacent tissue in TMA cohort (n=56). The *P* value was calculated by two-tailed unpaired *t* test.

D. The Spearman correlation analysis between LINC01711 expression and FAP expression in TCGA-LUAD cohorts.

E. The Kaplan-Meier survival analysis of LUAD patients with high LINC01711 expression and low LINC01711 expression.

F. The expression of LINC01711 in GEPIA database.

G. GSEA analysis on RNA-sequence data of si-LINC01711 vs si-NC group. n=3 biological repeats.

H. According to the coding potential assessment tool (CPAT), PRIDE reprocessing 2.0, PhyloCSF score, the coding score of *LINC01711* was very low, which was consistent with the characteristics of lncRNAs.

I. Co-expression analysis in TCGA-LUAD tumors identified 108 genes that are significantly positively correlated with LINC01711 (Spearman r > 0.5, FDR < 0.05). Motif scanning of the LINC01711 promoter region (2000bp upstream of TSS) using JASPAR2022 identified 144 transcription factors with predicted binding motifs. Intersecting the two sets yielded two transcription factors (MEIS3 and TWIST1) that were both co-expressed with LINC01711 and predicted to bind its promoter.

J. The DNA binding motif of MEIS3 and TWIST1 predicted by JASPAR.

K. JASPAR predicted the LINC01711 promoter region in which the motif of MEIS3 and TWIST1 hits.

L. The mRNA expression of TWIST1 and MEIS3 in FAP^+^CAFs and FAP^-^ CAFs (n=3 biological repeats). The *P* value was calculated by two-tailed unpaired *t* test.

M. Schematic diagram of LINC01711 truncated body.

All the results were shown as mean ± S.E.M. * *P*≤0.05, ** *P*≤0.01, *** *P*≤0.001, **** *P*≤0.0001.

**Figure S3.**

A. The Spearman correlation analysis between LINC01711 expression and LDHA expression in TCGA-LUAD cohorts.

B-D. The effectiveness of si-FGFR1, si-JAK2 and si-HER2 was validated by RT-qPCR (n=3 biological repeats). The *P* value was calculated by two-tailed unpaired *t* test.

E. The Spearman correlation analysis between LINC01711 expression and FGFR1 expression in TCGA-LUAD cohorts.

F. The effectiveness of si-LINC01711 validated by RT-qPCR (n=3 biological repeats). The *P* value was calculated by two-tailed unpaired *t* test.

G. RT-qPCR indicated that altering LINC01711 expression would not affect FGFR1 expression (n=3 biological repeats). The *P* value was calculated by two-tailed unpaired *t* test.

H. The effectiveness of LDH inhibitor was validated by LDHA activity assay (n=3 biological repeats). The *P* value was calculated by two-tailed unpaired *t* test.

I. RT-qPCR indicated that altering FGFR1 expression would not affect LINC01711 expression (n=3 biological repeats). The *P* value was calculated by two-tailed unpaired *t* test.

J. RT-qPCR indicated that applying LDH inhibitor would not affect LINC01711 expression (n=3 biological repeats). The *P* value was calculated by two-tailed unpaired *t* test.

K. RT-qPCR indicated that LINC01711 expression showed no significant difference between LDHA transfection and LDHA Y10F transfection group (n=3 biological repeats). The *P* value was calculated by two-tailed unpaired *t* test.

L. RT-qPCR indicated that the expression level of LDHA exogenously transfected was much higher than that of endogenous LDHA in FAP^+^ CAFs.

All the results were shown as mean ± S.E.M. * *P*≤0.05, ** *P*≤0.01, *** *P*≤0.001, **** *P*≤0.0001.

**Figure S4.**

A-E. ECAR assay, glucose uptake assay, lactate production assay and LDHA activity assay were performed in FAP^+^ CAFs transfected with si-LINC01711 or treated with LDH inhibitor. (n=3 biological repeats). The *P* value was calculated by two-tailed unpaired *t* test.

F. LDHA activity assay was performed in PBMCs transfected with si-LINC01711 or si-NC. (n=3 biological repeats). The *P* value was calculated by two-tailed unpaired *t* test.

All the results were shown as mean ± S.E.M. * *P*≤0.05, ** *P*≤0.01, *** *P*≤0.001, **** *P*≤0.0001.

**Figure S5.**

A. Schematic diagram of synthetic construct.

B. The expression of LINC01711 in FAP^+^ CAFs, FAP^-^ CAFs and other cells. n=3 biological repeats. The *P* value was calculated by two-tailed unpaired *t* test.

C. The effectiveness of SC01711 validated by RT-qPCR. n=3 biological repeats. The *P* value was calculated by two-tailed unpaired *t* test.

D. The abundance of si-LINC01711 in the major organs in mice was quantified by RT-qPCR. n=4 biological repeats.

E. Tissue section followed by scanning indicated that the synthetic construct could effectively delivery si-LINC01711 to lung in mice. Scale bars: 2000 μm or 200 μm.

F. Tissue section followed by scanning indicated that the efficiency of synthetic construct delivering si-LINC01711 to major organs in mice. Scale bars: 1000 μm or 100 μm.

G. IVIS detection indicated the targeting efficiency of major organs in mice.

H. H&E staining of liver from treated and normal mice. Scale bars: 100 μm.

I. RNA sequencing results of lung tissues from normal mice and treated mice. n=3 biological repeats.

J-K. The total photon ﬂux (F) and body weight (G) in the first orthotopic mouse model were measured. (n=5 biological repeats, the *P* value was determined by two-way ANOVA with Tukey’s multiple comparison test).

L-M. The total photon ﬂux (H) and body weight (I) in the second orthotopic mouse model were measured. (n=5 biological repeats, the *P* value was determined by two-way ANOVA with Tukey’s multiple comparison test).

All the results were shown as mean ± S.E.M. * *P*≤0.05, ** *P*≤0.01, *** *P*≤0.001, **** *P*≤0.0001.
