# Supplementary material for: Targeting LINC01711 in FAP+ cancer-associated fibroblasts overcomes lactate-mediated immunosuppression and enhances anti-PD-1 efficacy in lung adenocarcinoma
Source: Cell Death Dis. 2025 Aug 25;16(1):642. doi: 10.1038/s41419-025-07974-6 (PMC12379239; doi:10.1038/s41419-025-07974-6)
Supplement: Supplementary file 7 — Full and uncropped western blots. [file 41419_2025_7974_MOESM7_ESM.pdf]

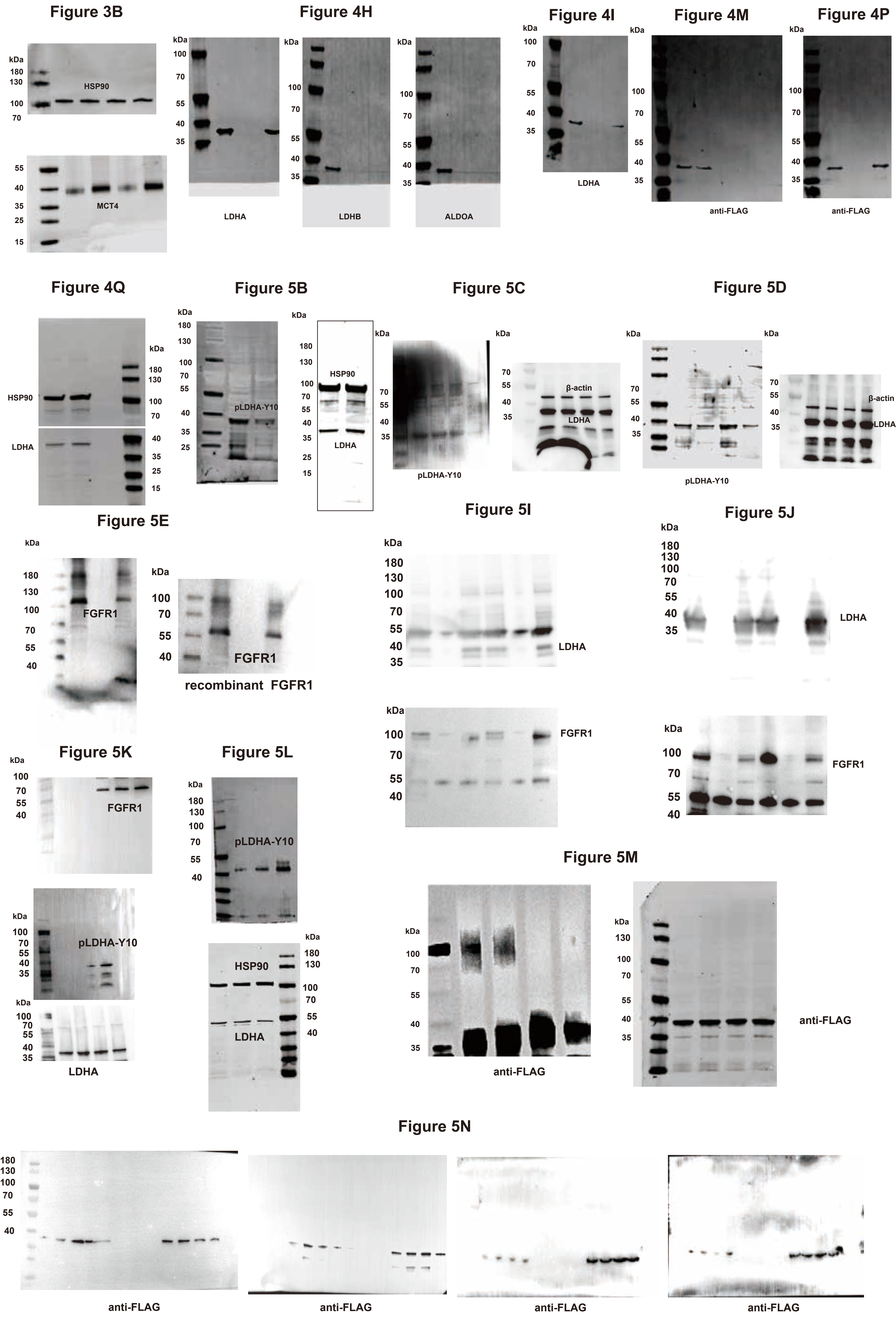
Figure 3B
kDa
180
130
100
70
HSP90
55
40
35
25
15
MCT4
Figure 4H
kDa
100
70
55
40
35
LDHA
kDa
100
70
55
40
35
LDHB
kDa
100
70
55
40
35
ALDOA
Figure 4I
kDa
100
70
55
40
35
LDHA
Figure 4M
kDa
100
70
55
40
35
anti-FLAG
Figure 4P
kDa
100
70
55
40
35
anti-FLAG
Figure 4Q
kDa
180
130
100
70
40
35
25
15
HSP90
LDHA
Figure 5B
kDa
180
130
100
70
55
40
35
25
pLDHA-Y10
kDa
180
130
100
70
55
40
35
25
15
HSP90
LDHA
Figure 5C
kDa
70
55
40
35
pLDHA-Y10
kDa
70
55
40
35
β-actin
LDHA
Figure 5D
kDa
70
55
40
35
pLDHA-Y10
kDa
70
55
40
35
β-actin
LDHA
Figure 5E
kDa
180
130
100
70
55
40
FGFR1
kDa
100
70
55
40
FGFR1
recombinant FGFR1
Figure 5I
kDa
180
130
100
70
55
40
35
LDHA
kDa
100
70
55
40
FGFR1
Figure 5J
kDa
180
130
100
70
55
40
35
LDHA
kDa
100
70
55
40
FGFR1
Figure 5K
kDa
100
70
55
40
FGFR1
kDa
100
70
55
40
35
pLDHA-Y10
kDa
100
70
55
40
35
LDHA
Figure 5L
kDa
180
130
100
70
55
40
pLDHA-Y10
kDa
180
130
100
70
55
40
HSP90
LDHA
Figure 5M
kDa
100
70
55
40
35
anti-FLAG
kDa
130
100
70
55
40
35
anti-FLAG
Figure 5N
180
130
100
70
55
40
anti-FLAG
anti-FLAG
anti-FLAG
anti-FLAG
